# Dataset of the suitability of major food crops in Africa under climate change

**DOI:** 10.1038/s41597-024-03118-1

**Published:** 2024-03-14

**Authors:** Abel Chemura, Stephanie Gleixner, Christoph Gornott

**Affiliations:** 1 Faculty of Geo-Information Science and Earth Observation (ITC), University of Twente, Enschede, The Netherlands; 2 Potsdam Institute for Climate Impact Research (PIK), Member of the Leibniz Association, Potsdam, Germany; 3 Agroecosystem Analysis and Modelling, Faculty of Organic Agricultural Sciences, University of Kassel, Kassel, Germany

**Keywords:** Climate-change impacts, Climate and Earth system modelling, Climate-change ecology

## Abstract

Understanding the extent and adapting to the impacts of climate change in the agriculture sector in Africa requires robust data on which technical and policy decisions can be based. However, there are no publicly available comprehensive data of which crops are suitable where under current and projected climate conditions for impact assessments and targeted adaptation planning. We developed a dataset on crop suitability of 23 major food crops (eight cereals, six legumes & pulses, six root & tuber crops, and three in banana-related family) for rainfed agriculture in Africa in terms of area and produced quantity. This dataset is based on the EcoCrop model parameterized with temperature, precipitation and soil data and is available for the historical period and until mid-century. The scenarios used for future projections are SSP1:RCP2.6, SSP3:RCP7.0 and SSP5:RCP8.5. The dataset provides a quantitative assessment of the impacts of climate change on crop production potential and can enable applications and linkages of crop impact studies to other socioeconomic aspects, thereby facilitating more comprehensive understanding of climate change impacts and assessment of options for building resilience.

## Background & Summary

Agriculture holds immense importance on the African continent, playing a vital role in its economy, food security, and livelihoods. Over sixty percent of Africa’s economically active population works directly in the agriculture sector for their sustenance and income with a significant share further employed downstream along the agricultural value chain^1–3^. Furthermore, agriculture serves as a source of raw materials for industries, contributes to foreign exchange earnings through exports, and supports rural development. In this regard, several crops are crucial for food security and economic wellbeing. Climate change has already and is projected to have significant impacts on food crops and farming systems in Africa from rising temperatures, changes in precipitation amounts, intensity and distribution, and increased frequency of extreme weather events, which alter the conditions necessary for successful crop growth. Studies have already shown that many regions are experiencing shifts in crop suitability, with traditional crops and agricultural areas becoming less viable and new areas emerging as potential production areas^4,5^. Both changes in temperature and precipitation conditions are responsible for these changes in potential production areas. Increasing temperatures due to climate change is pushing production areas beyond the temperature tolerance of many crops and increase heat stress^6,7^. Increasing temperatures also indirectly influence canopy level water balance by increasing plant water demand, reducing photosynthesis and accelerating evaporative losses^8,9^.

Climate change is also altering precipitation patterns, causing decreased precipitation and longer dry seasons in some regions, making it difficult for crops to obtain the necessary water for growth. This leads to reduced yields and even crop failures, as water stress becomes a critical limiting factor^10–12^. Conversely, other regions may face increased rainfall intensity and flooding, which can cause soil erosion, nutrient leaching, and waterlogging, making it also challenging to cultivate certain crops efficiently^13^. Therefore, changes in both temperature and precipitation under climate change disrupt established farming practices, threaten livelihoods, and exacerbate food insecurity. This is because crops that were once well-adapted to local climates may no longer thrive under the new conditions, leading to decreased agricultural production outcomes particularly in vulnerable communities that rely heavily on rainfed agriculture for sustenance and income^14^.

Despite the convincing evidence and consensus that climate change is impacting agriculture in Africa, there is a general lack of comprehensive data of climate change impact assessments for the continent. This is because current climate change impact studies are based on individual crop assessments at plot, region or country scale using different modelling approaches, climate data and assumptions which make standardized comparisons between space and time difficult or impossible. Many simulation results are scattered across the continent with primary data driving the analysis not readily available for potential users. To solve this problem, there have been regional and global efforts to provide easily accessible data and model results on which agricultural resilience can be built on. These include the Global Agro-Ecological Zones (GAEZ) approach by the Food and Agriculture Organization (FAO)^15^, decision-rule based approaches^16^ and global crop model assessments and improvement intercomparisons efforts such as those under AgMIP^17^ among other research efforts.

While the data, standardised protocols and outputs from these efforts are important for making informed decisions which increase climate resilience, there is need for more up-to-date studies on crop suitability, climate impacts and yield dynamics that are focused to the peculiar conditions, production systems and capacities in Africa. For example, while GAEZ provides suitability maps for 53 crops across the world, (i) as a global assessment, the data is likely constrained by conditions elsewhere in the world^15^ which may overestimate or underestimate the results for Africa, (ii) there are no explicit information about how each determined suitability matches the known production areas in Africa for building confidence in the data and for understanding the propagated uncertainties when such data are used and (iii) climate data used are not the most recently available dataset in line with recent model developments such as those used in the IPCC AR6. In addition, climate impact studies including those that use suitability models are currently heavily biased towards major cereals such as maize and sorghum, and to a lesser extent on cassava. This is limiting in two ways; (i) they do not capture the normal food basket of an average family that often comprises various crop products and (ii) suitability models run for one or two crops are not useful in showing potential agricultural transformation pathways such as ‘new’ or alternative crops that are possible for a site for crop switching or for diversifying crop choices.

To fill this gap, regional, or country or sub-national specific crop suitability studies have been conducted to identify climate impact hotspots, understand drivers of impact and inform adaptation planning^4,18–20^. Therefore, there is a current gap in studies as available evidence is either local at country level or from global studies, with dedicated continental studies missing. The information from crop suitability studies is so important in climate risk assessments for agriculture that it forms an important methodological pillar of the most recent IPCC ARC report on climate change impacts on food, fibre and other ecosystem products^21^. However, underlying data from the majority of the studies are not readily available for other researchers and analysists to use or link with their studies. Therefore, with the urgent need for action to mitigate climate change and adapt to its challenges on the agriculture sector, there is need to develop actionable and policy-relevant quantitative information on crop impacts in a standardized, open and well-documented database of crop suitability for major food crops in Africa under climate change. This database, in addition to identifying impacts and impact hotspots for specific crops and crop combinations, is important for those interested in analysing transformational pathways for agriculture, linking impacts to other socio-economic indicators such as conflict and/or as input in other models that may require such input.

To address this pertinent need, we developed a database of crop suitability across Africa for 23 crops that are important for food security, livelihoods and economic development under historical and future climatic conditions and scenarios. These crops are grouped as cereals (eight crops), legumes and pulses (six crops), root and tuber crops (six crops) and banana and related crops (three crops). These crops were selected based on their relevance for food security in Africa as indicated by harvested area, production output and utilisation in diets. The database uses both a standardized method and climate data to enable comparison across space and time.

## Methods

### The crop suitability concept

Crop suitability is a measure of the ability of climatic and other biophysical characteristics of an area to sustain a crop production cycle to meet current or expected targets^22^. It is a score of climatic feasibility for agronomic use of an area based on edapho-climatic and other variables for current or future conditions. Crop suitability models utilize associations between environmental variables and crops to identify environmental conditions within which crops can be grown on the basis of crop ecophysiology. The spatial distribution of environmental conditions that are suitable for the crops can then be estimated across a study region. Thus, crop suitability models characterize the environmental conditions in which a crop can be grown and assume that it will thrive in similar environments. The IPCC AR6 report^21^ describes this approach as models that compare the known climate suitability of species and habitats with projected climate conditions across different locations. Crop suitability models and their variants have been widely used in climate change impact studies at various scales and time periods across the world^23–29^.

### The EcoCrop model

We used the EcoCrop model, which is a generic mechanistic model which uses climate and soil data as inputs to determine the main niche to grow a crop and produces a suitability index depending on how far the conditions are met. The EcoCrop model was originally developed using the FAO-EcoCrop database of crop requirements and ranges^30^. The model uses monthly precipitation and temperature data together with soil pH to assess suitability at a grid scale for each crop. It uses the EcoCrop database parameters that correspond a crops’ required temperature and total rainfall ranges, and the minimum length of the crop specific growth cycle. For each grid site, the model simulates different possible growing seasons and selects the most suitable, starting at the beginning of each of the 12 months in a year, with the duration being determined by established growing length of the crop in the EcoCrop database. The technical implementation of the model has already been described in detail in literature^25,31,32^.

We selected EcoCrop because this crop suitability model requires few crop-specific parameters, it can be applied to many crops, including those where less detailed ecophysiological information is available and its outputs, to a large extent, match those from models requiring more complex processes^33^. The assumption is that the farmers are planting the crops during the seasons that are most suitable for the crop and the model does not account for double cropping within a year as only one most suited growing period is selected. Although double cropping is practiced in some regions across Africa, the biggest proportion of yield and production is obtained from the most optimal of the two seasons and therefore we capture the most important cropping cycles across the continent. EcoCrop takes into account the necessary genotypic variation without providing the detailed variety-level information except for specific crops such as wheat and rice with specific sub-types^34^. Therefore, we have confidence that EcoCrop is a reliable tool for assessing, quantifying and mapping the current and future suitability of important food crops in Africa by 2050. EcoCrop produces suitability values which range from 0 (unsuitable) to 1 (highly suitable).

### Climate and soil data

For model calibration we used the W5E5 observational data^35–37^, which integrates simulations from global weather models, satellite observations, and weather station observations. The dataset is at daily temporal resolution and the entire globe at 0.5°× 0.5° (~55 km × 55 km) grid spacing. W5E5 data was compiled to support climate bias adjustment of those climate models (General Circulation Models, GCMs) used in phase 3b of the Inter-Sectoral Impact Model Intercomparison Project (ISIMIP)^36,37^. Rainfall monthly sums were calculated from the daily data while for temperature, monthly averages were calculated since EcoCrop uses monthly data. Soil pH is another important parameter determining crop suitability in EcoCrop. The EcoCrop database provides numerical niche ranges for soil pH and these were included because soil pH is limiting in many areas in Africa, and influencing important crop processes such as water and nutrient uptake^38–40^. No reliable future soil pH projections were available and therefore we modelled with soil pH as static over time as we wanted only the impact of climate signal on suitability. Soil pH values were obtained from the ISRIC soil database^41,42^ and resampled with bilinear interpolation to match the resolution of the climate data. The final crop suitability for each cropland grid is obtained from the climatic and soil suitability values. The W5E5 data were used as observational dataset only for the model calibration process and the suitability data are based on historical and future projections from the ISIMIP3b climate data.

### The EcoCrop model calibration

Using the observational climate dataset (W5E5), some of the crops did not produce reliable suitability when compared with the reference validation data after initial model runs. This indicated that the default parameters were not sufficient to model these crops for Africa as they are generalized for the entire whole world. These crops were paddy rice, common wheat, cassava, white yam, plantain and banana. This was somewhat expected as these crops are mainly produced under different systems in South-East Asia and South America from which the parameters could be heavily influenced from. This meant that the model needed to be calibrated to match the production conditions in Africa. Initial analysis of the results showed that the area predicted as suitable was very small compared to the reference MAPSPAM data for these crops. Based on previous studies, we started the calibration by adjusting the rainfall parameters by decrements of 10 mm iteratively until the threshold accuracy is reached for that crop as rainfall is the most limiting factor for rainfed agriculture in Africa^43–45^. In cases where the crop did not reach 0.7 match with reference data (white yam and plantain), the maximum attainable rainfall parameters were retained, and temperatures similarly adjusted in a window by ±1 degree until ±5 for the ktmp and tavg parameters. In the calibration process we compared the result using an independent reference data obtained from thresholding the areas with less than the 25^th^ percentile of MAPSPAM yield as unsuitable^22^.

### Climate change projections

Using the calibrated model, the impact of climate change on crops suitability in Africa was assessed from projected climate and socio-economic conditions defined based on the combination of the Representative Concentration Pathways (RCPs) and the Shared Socio-economic Pathways (SSPs). RCPs are divided based on warming potential of emission levels. On the other hand, SSPs provide five distinct narratives about the future of the world, exploring a wide range of plausible trajectories of population growth, economic growth, technological development, trade development and implementation of environmental policies^46–48^. We selected a range from the most sustainable development (SSP1), regional rivalry (SSP3) and full fossil-fuelled development (SSP5) in linear combination with three RCPs.

A combination of RCPs and SSPs, therefore, represent more diverse future range of scenarios that are more probable by the integration of radiative forcing and socioeconomic development influences and provide the most current state of the art climate data from CMIP6^49^, providing a more comprehensive scenario matrix. This is because each SSP aligns broadly with one or two RCPs, allowing easy integration of SSPs and RCPs. The three combinations chosen were SSP1-RCP2.6 (SSP126), SSP3-RCP7.0 (SSP370) and SSP5-RCP8.5 (SSP585). Future climate projections were obtained from 10 GCMs of the ISIMIP3b, which use bias-adjusted and downscaled CMIP6 data. Historical simulations of ISIMIP3b cover 1850–2014, and future projections cover 2015–2100 at daily temporal resolution at 0.5° × 0.5° spatial grid. Our historical period is 1984 to 2014 while future projections are averages of data from 2035 to 2065. The 10 GCMs used are GFDL-ESM4, IPSL-CM6A-LR, MIROC6, MPI-ESM1–2-HR, MRI-ESM2–0, CanESM5, CNRM-CM6-1, CNRM-ESM2-1, EC-Earth3 and UKESM1–0-LL^50^. For each of the 10 models, the three SSPs were used for the modelling, and this resulted in 30 model runs for each crop.

### Conversion to binary suitability

In addition to the suitability score data, we also provide binary data on suitability class for each of the crops under current and projected climatic conditions. The binary suitability is a conversion of the suitability scores to suitable and unsuitable using a threshold. It is important to properly determine the threshold at which a crop can be considered as suitable and unsuitable^51,52^. It is important to emphasize that the suitability index measure the easy or difficulty in a crop completing its entire production cycle at a particular location with ‘reasonable’ outcomes. This is not linearly correlated to yield or productivity. Thus, the suitability class shows potentially cultivable land for each crop, notwithstanding that a crop can be produced outside this range in marginal areas but with significant higher costs or technology requirements. We hold the view that errors of excluding a potentially suitable area as unsuitable (error of omission) and including an unsuitable area as suitable (error of commission) hold similar penalty in Africa because agriculture is still being developed with potential for new crops while it is also requiring huge investments that need to be properly allocated against competing needs. Therefore, we sought a threshold that is able to balance error of sensitivity (true-positive rate) and specificity (true negative rate) (Se=Sp).

**Fig. 1:**
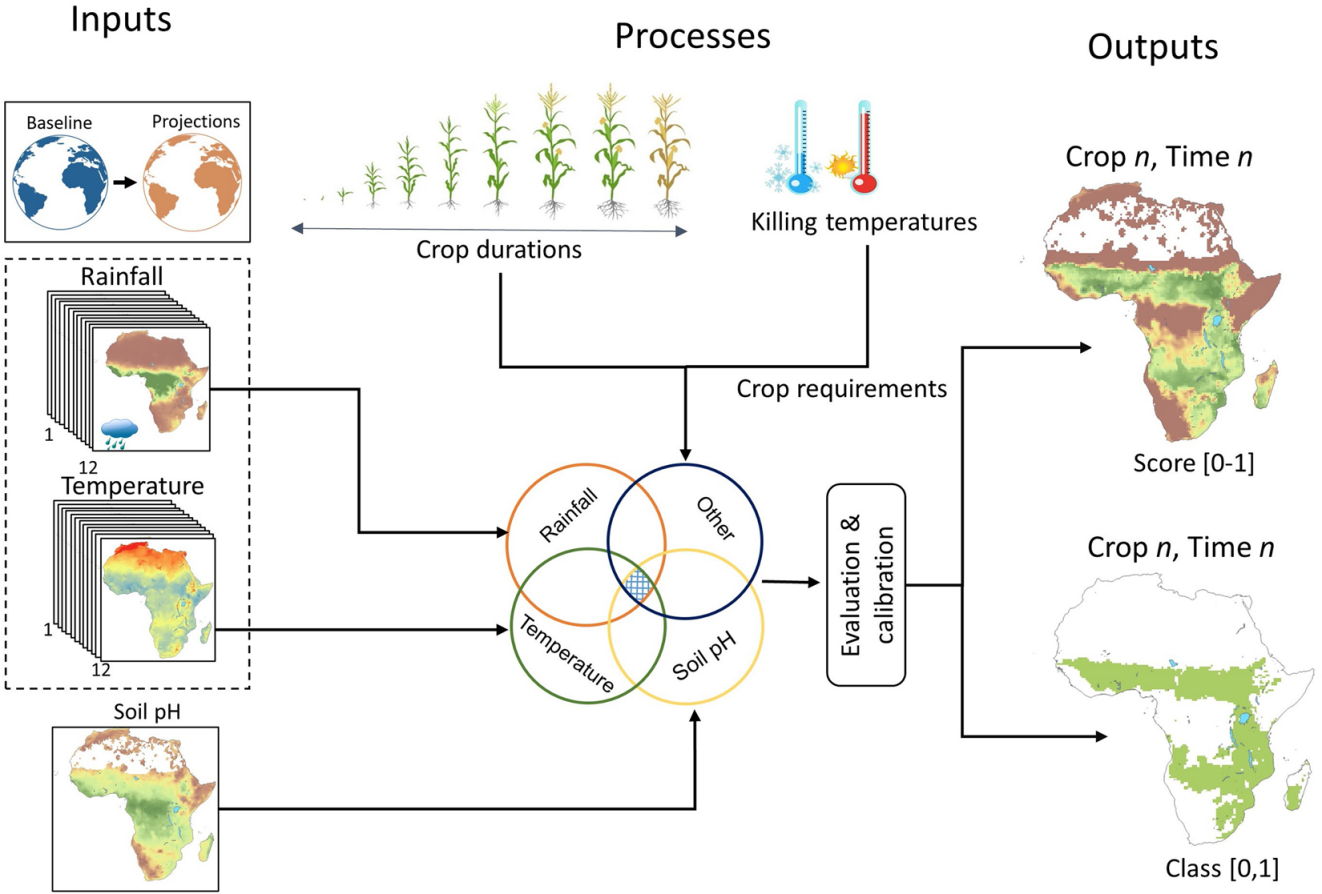
Schematic diagram showing the overview of the analysis framework. The agricultural suitability refers to land that is suitable for crop cultivation under the environmental conditions of each period and climate scenario.

The sensitivity-specificity equalization approach minimizes the absolute value of the difference between sensitivity and specificity and therefore ensures that the penalty of misclassification of both suitable areas and unsuitable areas is relatively the same^53–55^. This was considered important in this analysis because missing potentially suitable area (measured by sensitivity) reduce the area that can be considered in agricultural planning which is costly when the data are applied as alternative land will need to be used. On the other hand, erroneously including potentially unsuitable areas as suitable (measured by specificity) will also result in maladaptation outcomes as the included areas will not be productive, resulting in lost investments. Therefore both sensitivity and specificity are important measures of the model performance and therefore minimize uncertainties in practical application of the data for decision making in order to reduce possible penalties from both types of errors^55^. Figure 1 shows the schematic diagram of the workflow that was applied to produce the data.

## Data Records

**Fig. 2:**
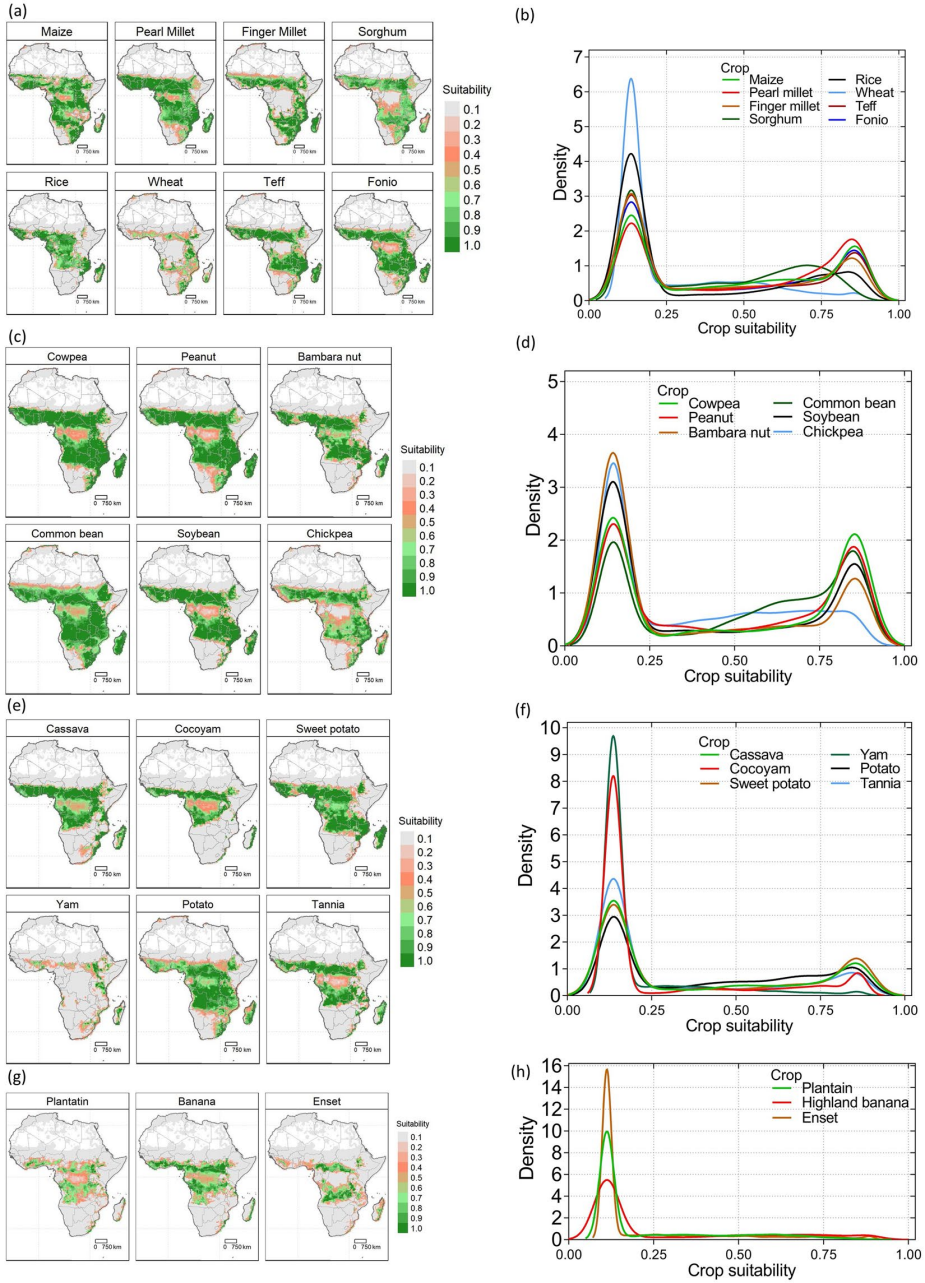
(**a**) Maps of crop suitability of cereals, (**b**) the density distributions cereals (**c**) maps of crop suitability of legumes and pulses, (**d**) the density distributions of legumes and pulses (**e**) maps of crop suitability of root and tuber crops, (**f**) the density distributions of root and tuber crops, (**g**) maps of crop suitability of banana and related crops and (**h**) the density distributions of banana and related crops under current climatic conditions in Africa.

**Fig. 3:**
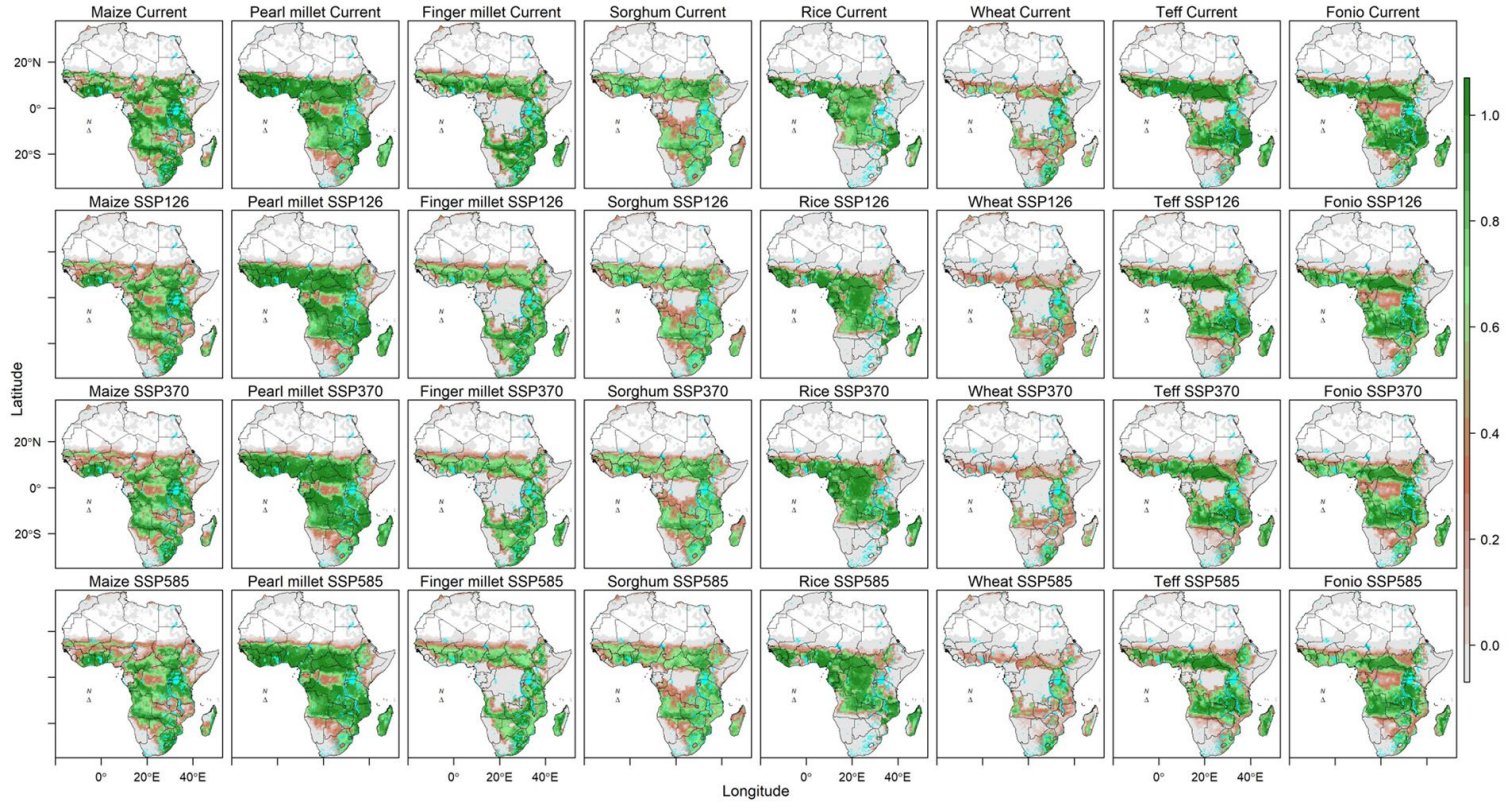
Suitability of 8 cereals crops in Africa under current and projected climatic conditions by 2050. The projections are a mean of 10 GCMS.

**Fig. 4:**
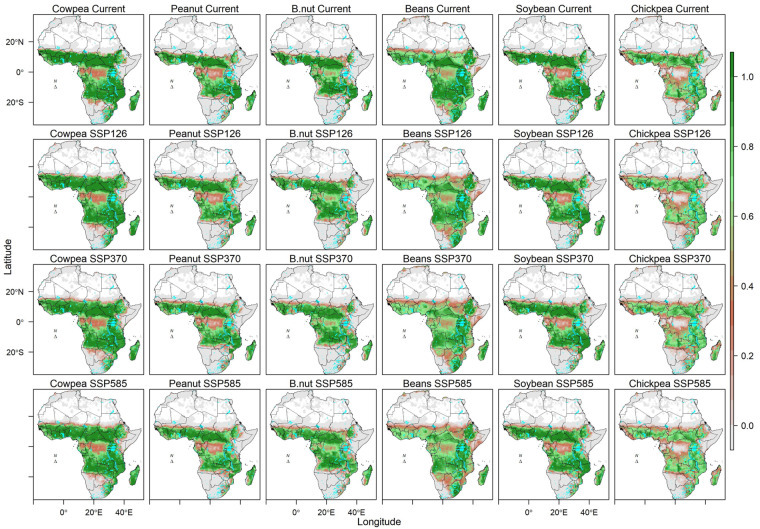
Suitability of 6 legume and pulses crops in Africa under current and projected climatic conditions by 2050. The projections are a mean of 10 GCMS.

**Fig. 5:**
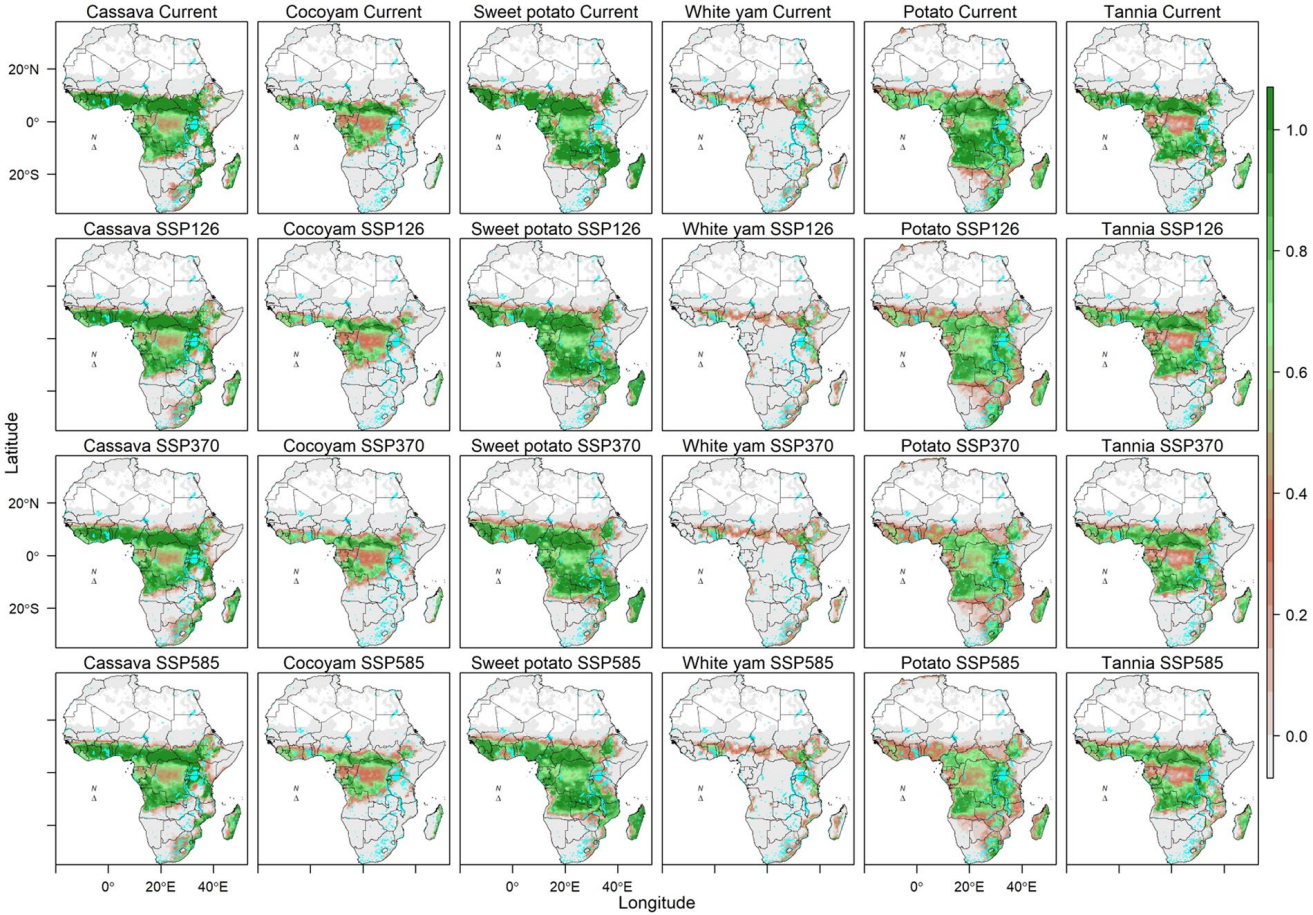
Suitability of 6 root and tuber crops in Africa under current and projected climatic conditions by 2050. The projections are a mean of 10 GCMS.

**Fig. 6:**
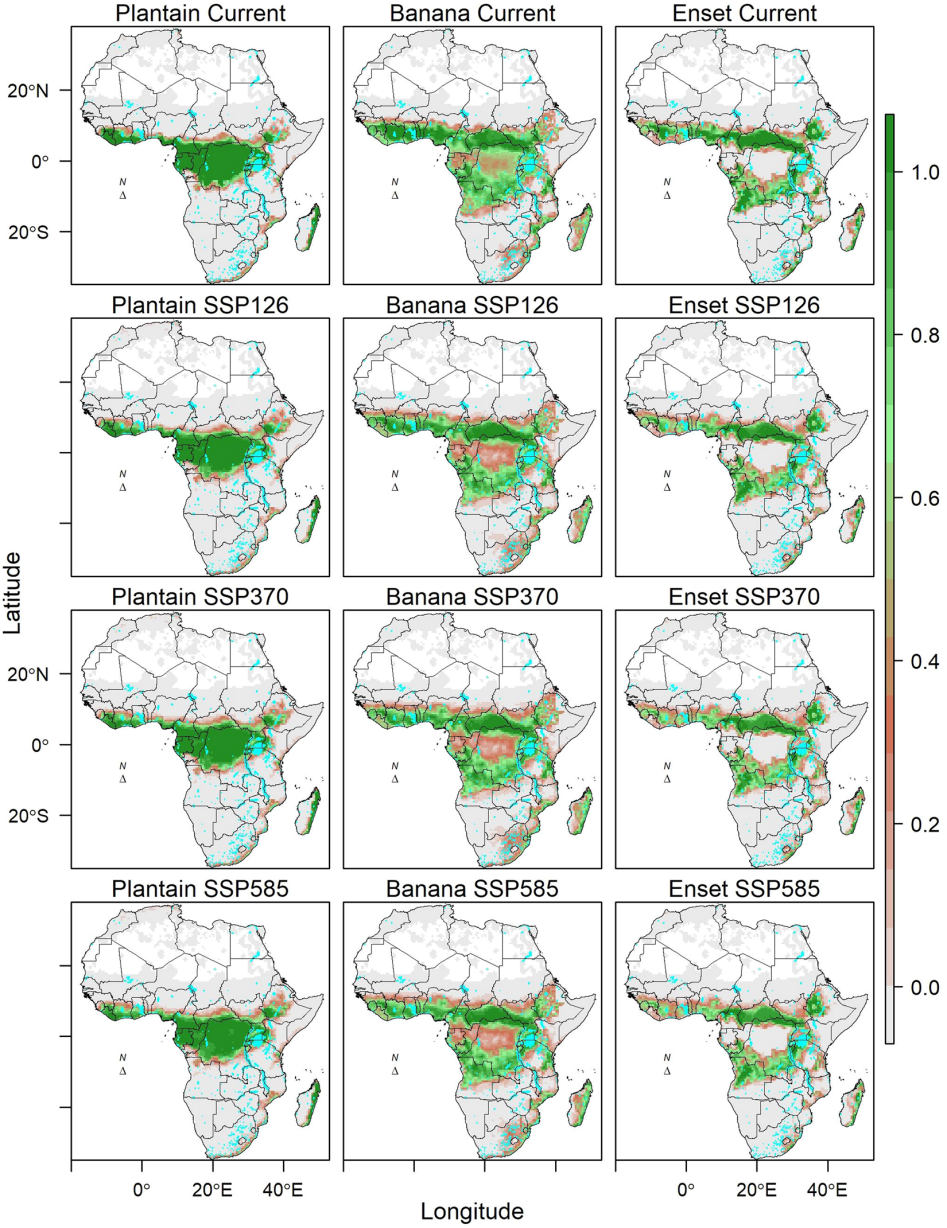
Suitability of three banana and related crops in Africa under current and projected climatic conditions by 2050. The projections are a mean of 10 GCMS.

**Fig. 7:**
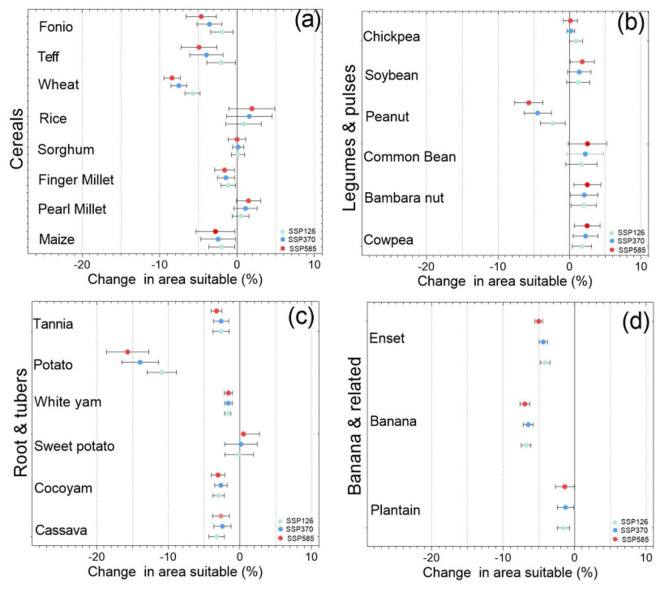
Percent area suitable for (**a**) cereals, (**b**) legumes and pulses, (**c**) root and tubers and banana and related crops in Africa under current and projected climatic conditions by 2050. The projections error bars show the variation while the colored dot is the mean of 10 GCMS.

The database contains suitability of 23 major crops (eight cereals, six legumes and pulses, six root and tuber crops, and three banana-related-related crops). The cereals included are maize, sorghum, pearl millet, finger millet, wheat, rice, teff and fonio. The legumes and pulses crops are cowpea, peanut, bambara nut, common bean, soybean and chickpea. The root and tuber crops are cassava, cocoyam, sweet potato, white yam, potato and tannia. The banana-related family crops are highland bananas, plantains and enset. Each of these crops, general current importance and use are described in SI1. For each crop, the suitability score [0-1) and suitability class [0,1] are provided for current climate (Fig. 2) and for 2050 average climatic conditions for cereals (Fig. 3), legumes and pulses (Fig. 4), root and tuber crops (Fig. 5), and banana and related (Fig. 6) across Africa. For future projected suitability we provide the suitability score and class derived from the thresholds in the calibration with MAPSPAM data. These results are provided for each of the 10 GCMs used as well as the mean of the climate models. The changes in the area can therefore be calculated for each crop from the suitability maps (Fig. 7). The data are freely available from the Figshare Digital Repository^56^.

## Technical Validation

We evaluated the model for accuracy by comparing the binary suitable area with reference datasets. We developed and implemented a comprehensive evaluation framework of the produced suitability maps as this is important in building confidence in the produced crop suitability maps for further use of the data. The reference datasets were used for the calculation of six independent validation metrics that were used for technical validation of the data. These reference datasets and how they were used for validation are described below:

- Global Biodiversity Information Facility (GBIF, www.gbif.org)^57^: The GBIF facilitates open access to species data worldwide for scientific research, conservation and sustainable development with over one billion occurrence records of species. To evaluate our model, we extracted the report ‘occurrence’ of each crop from 2000 to match the baseline period and then compared that to the modelled suitable area for that crop. The points were randomly cleaned to have one point corresponding to the grid resolution. We calculated the GBIF detection accuracy as the proportion of GBIF points that fall in the suitable area out of the total GBIF points. The data sources for GBIF data are listed in SI3.
- FAOSTAT (https://www.fao.org/faostat/en/#data/QC)^58^: The statistical data provided by the Food and Agricultural Organization of the United Nations (FAOSTAT) are a comprehensive, up-to-date and reliable database of food and agricultural statistics produced through methodologies standardized across countries to facilitate between country comparisons. We used the variable harvested area which we compared with the modelled suitable areas for that crop. The harvested area data were averaged from 2000 to 2020 for each country. Three model evaluation metrics were derived for the FAOSTAT harvested area data are as follows:

- FAOSTAT detection accuracy: This is defined as the correspondence of reported suitable area larger than one grid cell (0.5 × 0.5 degrees) in a country where the crop is reported as being produced. The basis for this evaluation is that (i) crops are reported/produced in the countries in which they are suitable, (ii) crops may be suitable in countries where they are not produced and (iii) crops are not supposed to be produced in countries in which they are not suitable unless under irrigation or greenhouse conditions. There was no information about irrigated areas for the crops in the countries and therefore that may bias the evaluation but only slightly since most of the crops are anyway produced under rainfed conditions in Africa. This is therefore a measure of proportion of the countries where the modelled suitability/unsuitability matched the reported production/non-production area of the crop.
- FAOSTAT correlation: We correlated the FAOSTAT harvested area in each country against the suitable area modelled for all countries in Africa. The basis of this validation method was that the countries with the most suitable areas are likely to have the largest harvested areas for those crops as farmers mostly produce crops in the areas that they are mostly suitable. However, the harvested areas could be biased by such factors as experience in the crop, markets, and culinary preferences to limit crop production in the areas that they are grown and therefore we are only accepting a positive correlation as acceptable model performance.
- Modelled suitable area > FAOSTAT harvest area: We assessed the proportion of the countries where the modelled suitable areas were larger than the reported FAOSTAT harvested area for each specific crop. The basis for this comparison was that EcoCrop predicts ‘absolute suitability’ which indicates where a species can be grown without major environmental constraints^59^. Therefore, the harvested area is the ‘realized niche’ for the crop which should always be lower than the ‘fundamental niche’ of the crop modelled by EcoCrop.

- Spatial Production Allocation Model (MAPSPAM v2r1, by HarvestChoice, https://mapspam.info/data/)^60^: We also evaluated the model using the data on harvested areas for each crop from MAPSPAM. The MAPSPAM data has been widely used in gridded modelling studies across the world because it provides farming system specific yields from a collection of sub-national statistical data integrating ancillary information including crop prices, population density and crop-specific biophysical suitability to distribute sub-national statistics within the cropland extent using cross entropy^61,62^. A total of 18 crops out of the 23 modelled were available in the MAPSPAM data for 2017. We derived two evaluation metrics from MAPSPAM data as follows:

- MAPSPAM area correlation: We correlated the MAPSPAM harvested suitable area modelled for all countries in Africa. Like for the FAOSTAT data, the basis was that the countries with the most suitable areas are likely to have the largest harvested areas for those crops as farmers mostly produce crops in the areas that they are mostly suitable.
- Model suitable area > MAPSPAM area: We assessed the proportion of the countries where the modelled suitable areas were larger than the MAPSPAM harvested area for each crop. The basis of this is similar to that used for comparing with FAOSTAT area.

**Fig. 8:**
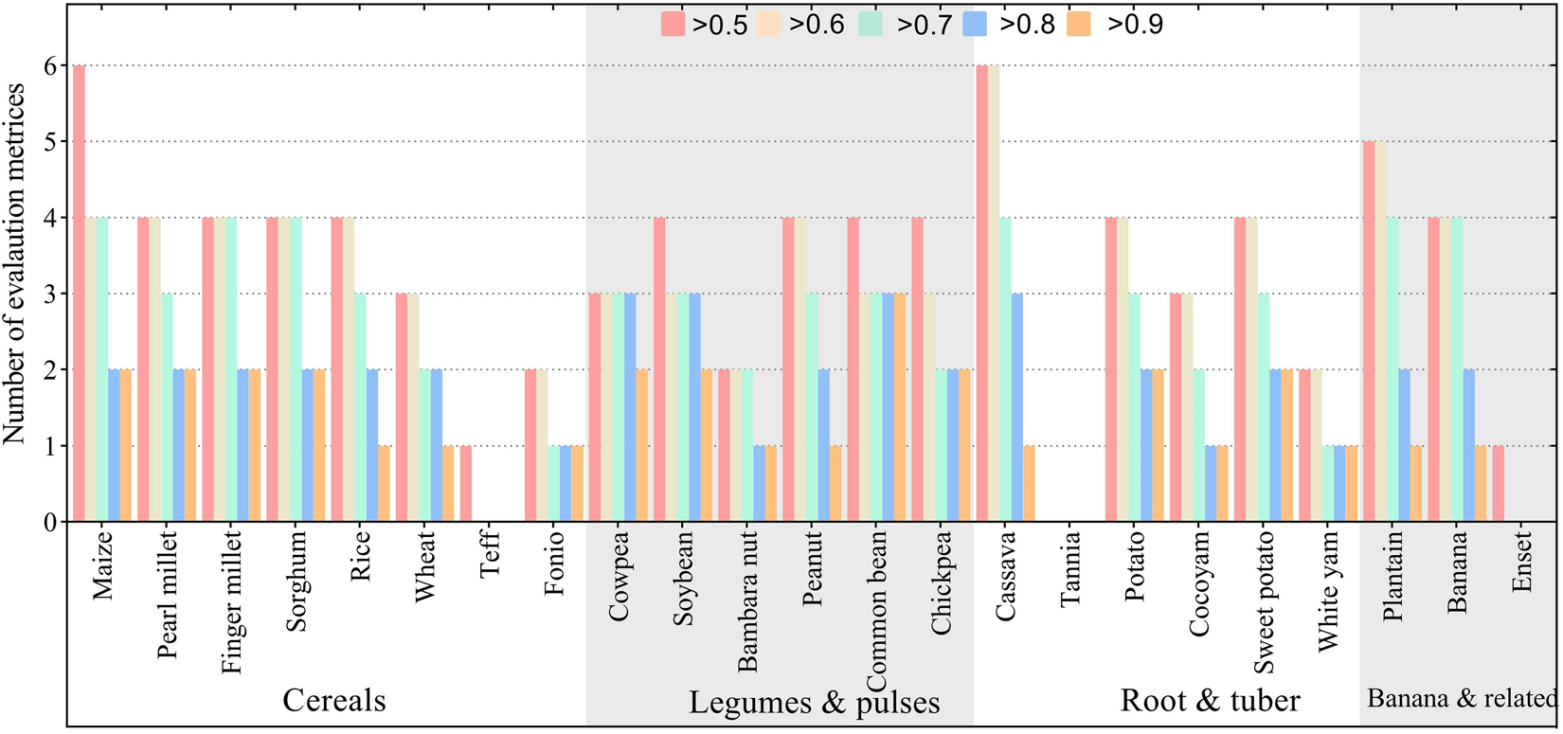
The number of times each crop exceeded the accuracy/correlation threshold between 0.5 and 0.9. The maximum possible times a threshold can be exceeded is 6 because 6 metrics are used. For example, for maize, the results show that evaluation metrics were above 0.5, 4 out of 6 evaluation metrics were above 0.7, and 2 out of the 6 were above 0.9.

**Fig. 9:**
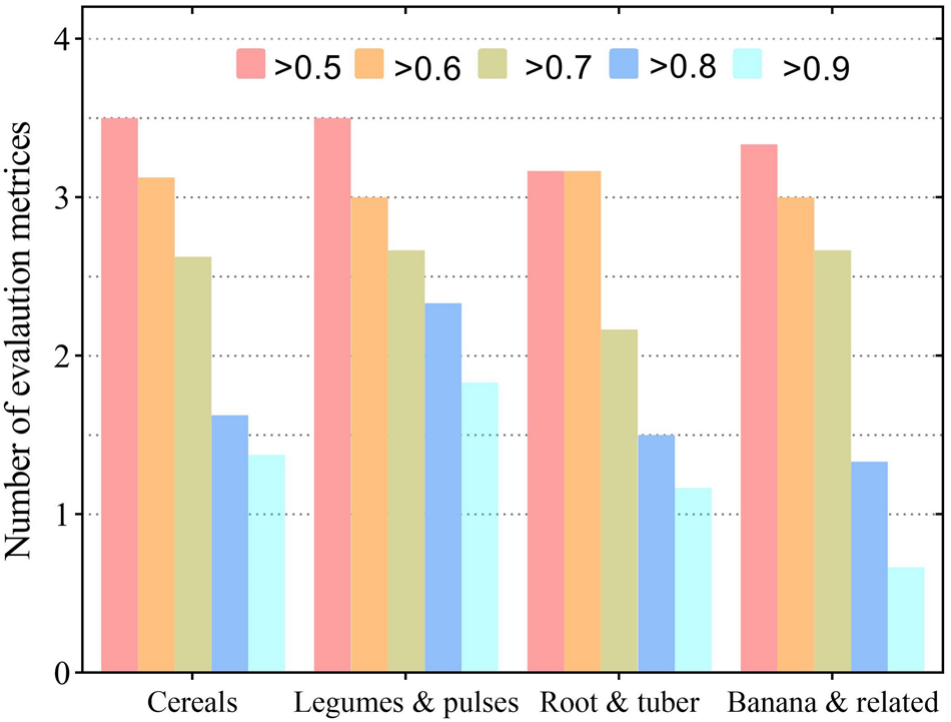
The mean times each crop class exceeded the accuracy/correlation threshold between 0.5 and 0.9. The maximum possible is 6 where all metrics are achieved in the evaluation.

The performance metrics for each crop and crop class are shown in SI2. From the assessment of the model evaluation metrics, we conclude the produced suitability maps are reliable to an acceptable/satisfactory level. This is because most of the crops score very high in at least 3 of the 6 evaluation metrics with at least a score of 0.7 except for teff, tannia and enset which had data gaps and were not available in most reference datasets (SI2, Fig. 8). Maize, cassava and plantain were consistent across evaluation metrics, with maize and cassava having score above 0.5 across all the 6 evaluation metrics, while for plantain it scored at least 0.5 in 5 of the 6 metrics used (Fig. 8). In terms of crop class, the model performance evaluation showed consistent results across the three classes (Fig. 9). The mean number of metrics that was exceeded was generally similar across crop classes. On average, legumes and pulses were more accurately modelled with an average of two crops exceeding 0.8 in accuracy metrics.

## Usage Notes

We provide two datasets: (i) crop specific suitability between 0 and 1 and (ii) binary suitability for suitable and unsuitable by converting the suitability index according to a threshold from the calibration [0,1]. For future projections we provide the mean of the 10 GCMs for each crop by 2050. Crop suitability data provide a narrative of when, where, and what type of crop can potentially grow under different GHG emissions and climate scenarios. They can provide quantitative information on the need for adaptation from analyzing the impacts and give indications of specific area that need to be targeted. The limit of using this dataset is endless depending on the applications and creativity of potential users and these are not prescribed. Users of this data, however, should be aware that there are some limitations inherent in crop suitability assessments. These include the fact that crop suitability assessments do not capture the effect of sudden or extreme events (heat waves, heavy precipitation events, dry spells etc.) as the data used are averaged over a month. In addition, climate change may influence crop suitability in a more complex way than captured by the used climatic parameters. For example, new pests or diseases or CO_2_ fertilisation effects can influence crop suitability under climate change. Although the role of climate is known to be important for rainfed agriculture across Africa, land use systems, cultural aspects and access to resources may also influence agricultural production. In addition, not all area considered suitable may not be available for use for agricultural purposes as it includes other such areas as protected area, urban areas and other non-available land at the resolution of the pixels used.

The descriptions of the abbreviations used for each of these are given in Table 1. The file structure also corresponds to the naming convention used starting with crop type (Banana, Cereals, Legumes and Roots), data type (Suit for suitability score and Suit_cl for suitability class), climate scenario (Current, SSP126, SSP370 and SSP585), and for projections processing level (GCM for raw data and Mean for mean of the models).) We therefore provide 1,394 data points in this database from the combination of the 23 crops, two data types, four scenarios, 10 climate model and additional statistical data from the mean of the models.

**Table 1:** Description of abbreviations and nomenclature used in archiving the data.

Code	Full name	TypeT	Code	Full name	TypeT
Cereals	Cereals	Class	sweetpotato	Sweet potato	Crop
Pulses	Legumes & pulses	Class	whiteyam	White yam	Crop
Roots	Root & tubers	Class	banana	Banana	Crop
Banana	Banana & related	Class	enset	Enset	Crop
maize	Maize	Crop	plantain	Plantain	Crop
pmillet	Pearl millet	Crop	suit	Suitability [0-1]	Data type
fmillet	Finger millet	Crop	suit_cl	Binary suitability [0,1]	Data type
sorghum	Sorghum	Crop	curr	Current	Period
rice	Rice	Crop	s126	SSP1:RCP2.6	Scenario
wheat	Wheat	Crop	s370	SSP3:RCP7.0	Scenario
teff	Teff	Crop	s585	SSP5:RCP8.5	Scenario
fonio	Fonio	Crop	cane	CanESM5	Climate model
cowpea	Cowpea	Crop	cnrc	CNRM-CM6	Climate model
soybean	Soybean	Crop	cnrm	CNRM-ESM2-1	Climate model
bambnut	Bambara nut	Crop	erth	EC-Earth3	Climate model
peanut	Peanut	Crop	gfdl	GFDL-ESM4	Climate model
commbean	Common bean	Crop	ipsl	IPSL-CM6A-LR	Climate model
chickpea	Chickpea	Crop	miro	MIROC6	Climate model
cassava	Cassava	Crop	mpie	MPI-ESM1-2-HR	Climate model
tannia	Tannia	Crop	mrie	MRI-ESM2-0	Climate model
potato	Potato	Crop	uksm	UKESM1-0-LL	Climate model
cocoyam	Cocoyam	Crop	mn	Mean	Statistical

These are also provided in the txt file accompanying that data.

### Supplementary information

Table 1

## Data Availability

Script files were created using the R statistical programming and are available with the data provided and on Github (https://github.com/achemura/cropsuitafriq/).
