# Supplementary material for: Dataset of the suitability of major food crops in Africa under climate change
Source: Sci Data. 2024 Mar 14;11:294. doi: 10.1038/s41597-024-03118-1 (PMC10940296; doi:10.1038/s41597-024-03118-1)
Supplement: Supplementary file 1 — Table 1 [file 41597_2024_3118_MOESM1_ESM.docx]

**Dataset of the suitability of major food crops in Africa under climate change**

Abel Chemura^1,2^, Stephanie Gleixner^2^ and Christoph Gornott^1,3^

^1^Faculty of Geo-Information Science and Earth Observation (ITC), University of Twente, Enschede, The Netherlands.

^2^Potsdam Institute for Climate Impact Research (PIK), Member of the Leibniz Association, Potsdam, Germany.

^3^Agroecosystem Analysis and Modelling, Faculty of Organic Agricultural Sciences, University of Kassel, Kassel, Germany.

Corresponding author: Abel Chemura (a.chemura@utwente.nl)

Contents

- SI1: Description of the 23 crops forming this database with information on their scientific name, botany and current use across Africa
- SI2: Skill scores of the suitability data for each crop calculated on the current suitability against reference data.
- SI3: Sources of GIBIF data that was used

### Supplementary information

**SI1: Description of the 23 crops forming this database with information on their scientific name, botany and current use across Africa. Information from EcoCrop^1^ and Garrity et al^2^**

| Class | Crop name | Botany, importance and current distribution |
| --- | --- | --- |
| Cereals | Maize (*Zea mays* (L.)) | A tall vigorous annual grass and grain crop. Maize is a major staple food crop grown in diverse agro-ecological zones and farming systems, and widely consumed in different forms of preparation. |
|  | Sorghum (*Sorghum bicolor* (L.) Moench) | A single-stemmed grass and cereal crop reaching a height of 1-4 m. Sorghum is the second most important cereal after maize and with increasing demand across the continent for both food and fodder. |
|  | Pearl millet (*Pennisetum glaucum* (L.)) | An erect, freely tillering, tufted grass and grain crop reaching a height of 0.5-4 m. Millet is highly nutritious and is highly resistant to droughts, requires less irrigation and other management than maize. |
|  | Finger millet (*Eleusine coracana* (L.)) | A grass and cereal crop reaching a height of 1 m or more. It is a short cycle crop that is increasingly popular for short duration cropping and drought tolerance. |
|  | Wheat (*Triticum durum* (Desf.) and *Triticum aestivum* (L.)) | It is a hexaploid annual grass growing up to 1.2 m tall. Wheat is widely grown in Africa and is a major food crop for several countries, although net demand is higher than production. |
|  | Rice (*Oryza sativa* (L.) s. japonica and *Oryza sativa* (L.) s. indica)) | It is an annual grass which has round jointed culm with long narrow leaves. Demand for rice is the fastest growing in Africa and yet production is far less than the growing demand and Africa is a net importer of rice. |
|  | Teff (*Eragrostis tef* (Zucc.)) | A grass and cereal crop with culms up to 1.2 m high. Teff is a grain mainly associated with the Horn of Africa countries, Ethiopia and Eritrea where it is an important crop. |
|  | Fonio millet (*Digitaria exilis* (Kippist)) | An erect, free-tillering grass and cereal crop reaching a height of about 0.45 m. It is consumed mainly in West African countries but increasingly becoming important for its drought tolerance. |
| Legumes and pulses | Cowpea (*Vigna unguiculata unguic.* (L.)) | It is a herbaceous, prostrate, climbing, or sub-erect to erect legume, growing 0.15-0.80 m high. Cowpea is one of the most important grain legumes in Africa. Its dry grain, fresh-shelled 'peas', fresh pods, and fresh and dried leaves and flowers are consumed. |
|  | Peanut (Arachis hypogaea (L.)) | An annual legume reaching a height of up to 0.6 m. Widely grown in many countries across Africa especially within smallholder farming systems as a cash and food crop. |
|  | Bambara nut (*Voandzeia subterranea* (L.)) | A heavily branched, creeping, herbaceous legume with trifoliate flowers and whitish-yellow flowers grown for its edible seeds. The seeds are eaten fresh, boiled, or canned and have a taste similar to the garden pea. It is indigenous to tropical West Africa. |
|  | Common bean (*Phaseolus vulgaris* (L.)) | A slightly pubescent, erect, herbaceous bush or twinning vine with well-developed tap-root. It is a major staple, and contributes to food and nutrition security as it is rich in protein and other nutrients. |
|  | Soybean (*Glycine max* (L.)) | A bushy herbaceous annual legume reaching a height of 0.20-1.80 m. Soybean is produced across Africa and the legume is the richest and cheapest sources of protein for human and animal consumption. |
|  | Chickpea (*Cicer arietinum* (L.).) | A branched, hairy, herbaceous legume reaching 0.20-1.00 m in height with straight or bent stems and roots reaching 1-2 m deep. This crop is produced mainly for human food and animal feeds. |
| Root and tuber | Cassava (*Manihot esculenta* Crantz.,) | An erect shrub with an upright woody stem reaching a height of 4 m or more. Cassava is a versatile crop that can be used as a staple food, livestock feed, or raw materials, and many parts utilized. |
|  | Cocoyam/Taro (*Colocasia esculenta* (L.)) | A herbaceous plant with a underground corm producing a few large leaves with long erect petioles. It is mainly cultivated for its tubers, which contain large quantities of small starch grains and are rich in protein, calcium, and phosphorus, but are low in fats and protein. |
|  | Sweet potato (*Ipomoea batatas* (L.)) | A herbaceous vine with a creeping growth habit that produce milky juice. Sweet potato is one of the most widely grown root crops in Africa. |
|  | White yam (*Dioscorea rotundata* (L.)) | Yams are large starchy tubers produced by annual and perennial vines that may reach a length of 2-30 m. Yams are primary agricultural commodities and major staple crops in west Africa. |
|  | Potato (*Solanum tuberosum* L) | This is a dicotyledonous tuberous herbaceous plant that can grow to about 0.60 m high. There is huge and growing demand for potato across Africa especially in central and eastern Africa. |
|  | Tannia (*Xanthosoma sagittifolium* (L.)) | A robust herbaceous plant with a short stem, on the apex of which are borne a few large leaves with long erect petioles. In tropical Africa tannia constitute an important, often major, components in traditional diets together with cocoyam. The tubers and the leaves are also eaten as part of diverse dishes. |
| Banana and related crops | Highland bananas (*Musa acuminata*) | It is the largest herbaceous flowering plant grow from a "corm". Bananas are important staple foods in many countries in Africa where they are eaten ripe, cooked, as flour, or chips. |
|  | Plantains (*Musa balbisiana*) | The plantain plant resembles the banana but the fruits of plantain are longer, have a thicker skin, and contain more starch. They are usually cooked and not eaten raw unless they are very ripe. Very important in the humid areas of West and Central Africa. |
|  | Enset (*Ensete ventricosum* (Welw.)) | A large monocarpic unbranched herb. The pseudostem is often markedly swollen at the base. The thickened pseudostems and swollen underground stems are eaten cooked or fermented, usually after it has been made into bread. They are commonly consumed in the horn of Africa. |

**SI2: Skill scores of the suitability data for each crop calculated on the current suitability against reference data.**

For the GBIF this is the proportion of points of observed crop points that are existing in the modelled suitable area for that crop. The FAOSTAT Detection is the proportion of countries that report a crop to that modelled. The FAOSTAT correlation is the correlation of modelled area and FAOSTAT harvested area for countries. Model > FAOSTAT is the proportion of countries where the modelled suitability is higher than the reported harvested area from FAOSTAT. The MAPSPAM correlation is the correlation of modelled area and MAPSPAM harvested area for countries. Model > MAPSPAM is the proportion of countries where the modelled suitability is higher than the reported harvested area from MAPSPAM.

| Crop type | Crop | GBIF  Detection | FAOSTAT  Detection | FAOSTAT correlation | Model > FAOSTAT | MAPSPAM Correlation | Model > MAPSPAM |
| --- | --- | --- | --- | --- | --- | --- | --- |
| Cereals | Maize | 0.72 | 0.78 | 0.53 | 1.00 | 0.59 | 0.95 |
|  | Pearl millet | 0.64 | 0.78 | 0.01 | 1.00 | -0.08 | 0.97 |
|  | Finger millet | 0.73 | 0.76 | 0.18 | 1.00 | 0.16 | 1.00 |
|  | Sorghum | 0.71 | 0.80 | 0.28 | 1.00 | 0.23 | 0.92 |
|  | Rice | 0.61 | 0.80 | 0.37 | 1.00 | 0.42 | 0.90 |
|  | Wheat | 0.18 | 0.70 | -0.12 | 1.00 | 0.31 | 0.81 |
|  | Teff | 0.58 | 0.36 | — | — | — | — |
|  | Fonio | 0.69 | 0.36 | 0.45 | 1.00 | — | — |
| Legumes & pulses | Cowpea | 0.88 | 0.42 | 0.04 | 1.00 | -0.02 | 0.93 |
|  | Soybean | 0.83 | 0.56 | 0.21 | 1.00 | 0.15 | 0.97 |
|  | Bambara nut | 0.80 | 0.38 | -0.13 | 1.00 | — | — |
|  | Peanut | 0.61 | 0.72 | 0.34 | 1.00 | 0.34 | 0.81 |
|  | Common bean | 0.99 | 0.56 | 0.28 | 1.00 | 0.24 | 0.95 |
|  | Chickpea | 0.55 | 0.34 | 0.70 | 1.00 | 0.27 | 0.93 |
| Root & tubers | Cassava | 0.81 | 0.78 | 0.64 | 1.00 | 0.61 | 0.89 |
|  | Tannia | 0.50 | 0.32 | — | — | — | — |
|  | Potato | 0.80 | 0.66 | 0.05 | 1.00 | 0.22 | 0.91 |
|  | Cocoyam | 0.46 | 0.76 | 0.06 | 1.00 | 0.22 | 0.67 |
|  | Sweet potato | 0.64 | 0.76 | 0.21 | 1.00 | 0.23 | 0.92 |
|  | White yam | 0.33 | 0.66 | 0.14 | 1.00 | 0.01 | 0.48 |
| Banana & related | Plantain | 0.7 | 0.76 | 0.36 | 1.00 | 0.72 | 0.86 |
|  | Banana | 0.74 | 0.74 | 0.20 | 1.00 | 0.14 | 0.89 |
|  | Enset | 0.55 | — | — | — | — | — |

**SI3: Sources of GIBIF data that was used**

1. Aboagye L, Osei-Kofi P, Owusu-Achiaw R (2022). Crop Wild Relatives (CWRs) Occurrence in Ghana. Version 1.2. Conservation Alliance International. Occurrence dataset https://doi.org/10.15468/pbn67c accessed via GBIF.org on 2022-09-04.
2. Aboagye L, Osei-Kofi P, Owusu-Achiaw R (2022). Crop Wild Relatives (CWRs) Occurrence in Ghana. Version 1.2. Conservation Alliance International. Occurrence dataset https://doi.org/10.15468/pbn67c accessed via GBIF.org on 2022-09-04.
3. Abrahamyan A (2022). Seed Collection of Crops and Crops’ Wild Relatives (CWRs) of Armenia. Version 1.1. "Scientific Center of Agrobiotechnology" Branch of ANAU Foundation. Occurrence dataset https://doi.org/10.15468/czqf4h accessed via GBIF.org on 2022-09-04.
4. Adam Mickiewicz University in Poznań Natural History Collections of the Faculty of Biology AMU Occurrence dataset https://doiorg/1015468/54hgbz accessed via GBIForg on 2022-09-04.
5. Adam Mickiewicz University in Poznań. Natural History Collections of the Faculty of Biology AMU. Occurrence dataset https://doi.org/10.15468/54hgbz accessed via GBIF.org on 2022-09-04.
6. AFFOUARD A, JOLY A, LOMBARDO J, CHAMP J, GOEAU H, CHOUET M, GRESSE H, BONNET P (2022). Pl@ntNet observations. Version 1.8. Pl@ntNet. Occurrence dataset https://doi.org/10.15468/gtebaa accessed via GBIF.org on 2022-09-04.
7. AFFOUARD A, JOLY A, LOMBARDO J, CHAMP J, GOEAU H, CHOUET M, GRESSE H, BOTELLA C, BONNET P (2022). Pl@ntNet automatically identified occurrences. Version 1.8. Pl@ntNet. Occurrence dataset https://doi.org/10.15468/mma2ec accessed via GBIF.org on 2022-09-04.
8. Ajikah L, Onuminya T, Ogundipe O, Akomaye F (2022): Biodiversity of Delta Coastal Environment, Delta State, Nigeria. v1.4. University of Calabar. Dataset/Occurrence. https://ipt-nigeria.gbif.fr/resource?r=floraofdeltacoastalenvironments&v=1.4 https://doi.org/10.15468/j9r2up accessed via GBIF.org on 2022-09-04.
9. AKE-ASSI E A, KOUASSI A F, YAO K, TRA BI B F, AKPATOU B, BARIMA S S Y, BAKAYOKO A, TIEBRE M S, OUATTARA D, N'Goran B (2020). Les spermaphytes de l'Herbier du Centre National de Floristique de Côte d'Ivoire: deuxième partie. Version 1.3. Centre National de Floristique. Occurrence dataset https://doi.org/10.15468/j4fnjr accessed via GBIF.org on 2022-09-04.
10. AKOEGNINOU A (2016). Herbier du Bénin. Version 1.2. GBIF Benin. Occurrence dataset https://doi.org/10.15468/uxi0h7 accessed via GBIF.org on 2022-09-04.
11. ALOHOU E, GBO F C, KOTCHOFA M (2019). "Répertoire des Forêts sacrées dans les Départements de l’Ouémé t du Plateau. Data mobilized in the framework of BID National project BID-AF2015-0065-NAC and funded by EU. Version 1.3. GBIF Benin. Occurrence dataset https://doi.org/10.15468/iozuua accessed via GBIF.org on 2022-09-04.
12. Aona L (2024). HURB herbarium - Herbário da Universidade do Recôncavo da Bahia - Herbário Virtual REFLORA. Version 1.1. Instituto de Pesquisas Jardim Botanico do Rio de Janeiro. Occurrence dataset https://doi.org/10.15468/mcq5wk accessed via GBIF.org on 2022-09-04.
13. Arsénio P, Cunha A R, Paes P, Vasconcelos T, Figueira R (2021). Herbário João de Carvalho e Vasconcellos, I.S.A./U.L. Instituto Superior de Agronomia / Universidade de Lisboa. Occurrence dataset https://doi.org/10.15468/olfpjv accessed via GBIF.org on 2022-09-04.
14. Asase A (2021). Grass Collections from Ghana. Version 1.4. Ghana Herbarium. Occurrence dataset https://doi.org/10.15468/yr8o3r accessed via GBIF.org on 2022-09-04.
15. Asase A (2021). Medicinal plants and associated plant collections at Ghana Herbarium. Version 1.1. Ghana Biodiversity Information Facility (GhaBIF). Occurrence dataset https://doi.org/10.15468/rti1bq accessed via GBIF.org on 2022-09-04.
16. Asase A (2021). Medicinal plants and associated plant collections at Ghana Herbarium. Version 1.1. Ghana Biodiversity Information Facility (GhaBIF). Occurrence dataset https://doi.org/10.15468/rti1bq accessed via GBIF.org on 2022-09-04.
17. Asase A (2021). Plants of Ghana. Version 1.1. Ghana Herbarium. Occurrence dataset https://doi.org/10.15468/e8rhqm accessed via GBIF.org on 2022-09-04.
18. ASSOGBA O N M (2020). Floristic diversity of potential exploitable ranches in the Zou and Collines departments of Benin. Laboratoire d'Ecologie Appliquée/Université d’Abomey-Calavi (LEA/UAC). Sampling event dataset https://doi.org/10.15468/vxe2v4 accessed via GBIF.org on 2022-09-04..
19. ASSOGBA O N M (2021). Floristic diversity of protected areas in the north of Benin. Laboratoire d'Ecologie Appliquée/Université d’Abomey-Calavi (LEA/UAC). Sampling event dataset https://doi.org/10.15468/g2tkwf accessed via GBIF.org on 2022-09-04.
20. Australia's Virtual Herbarium (2022) National Herbarium of Victoria (MEL) AVH data Occurrence dataset https://doiorg/1015468/rhzrxw accessed via GBIForg on 2022-09-04.
21. Australia's Virtual Herbarium (2022). National Herbarium of Victoria (MEL) AVH data. Occurrence dataset https://doi.org/10.15468/rhzrxw accessed via GBIF.org on 2022-09-04.
22. BAKAYOKO A, CHATELAIN C, KONE M W, KONE I, OUATTARA D, YAO K, GAUTIER L (2020). Occurrences des échantillons de plantes de l'Herbier du Centre Suisse de Recherches Scientifiques (CSRS) en Côte d'Ivoire. Version 1.2. Centre Suisse de Recherches Scientifiques en Côte d'Ivoire. Occurrence dataset https://doi.org/10.15468/gdbrsj accessed via GBIF.org on 2022-09-04..
23. Bijmoer R, Scherrenberg M, Creuwels J (2022) Naturalis Biodiversity Center (NL) - Botany Naturalis Biodiversity Center Occurrence dataset https://doiorg/1015468/ib5ypt accessed via GBIForg on 2022-09-04.
24. Biologiezentrum Linz Oberoesterreich. Biologiezentrum Linz. Occurrence dataset https://doi.org/10.15468/ynjblx accessed via GBIF.org on 2022-09-04.
25. Bioversity International SINGER Coordinator Occurrence dataset https://doiorg/1015468/oya7kn accessed via GBIForg on 2022-09-04.
26. Bioversity International The System-wide Information Network for Genetic Resources (SINGER) Occurrence dataset https://doiorg/1015468/uw1buh accessed via GBIForg on 2022-09-04.
27. Bioversity International. EURISCO, The European Genetic Resources Search Catalogue. Occurrence dataset https://doi.org/10.15468/a3lnmd accessed via GBIF.org on 2022-09-04.
28. Bioversity International. SINGER Coordinator. Occurrence dataset https://doi.org/10.15468/oya7kn accessed via GBIF.org on 2022-09-04.
29. Bissiengou P, Wieringa J, Creuwels J, Engone Obiang N L (2019). Herbier National du Gabon. Version 1.4. Herbier National du Gabon. Occurrence dataset https://doi.org/10.15468/sgxokl accessed via GBIF.org on 2022-09-04.
30. Borchsenius F (2022). The AAU Herbarium Database. Herbarium of the University of Aarhus. Occurrence dataset https://doi.org/10.15468/7uigwo accessed via GBIF.org on 2022-09-04.
31. Botanic Garden and Botanical Museum Berlin (2017). Herbarium Berolinense, Berlin (B). Occurrence dataset https://doi.org/10.15468/dlwwhz accessed via GBIF.org on 2022-09-04.
32. Botanischer Garten, TU Dresden. Herbarium Dresdense. Occurrence dataset https://doi.org/10.15468/qwjw7w accessed via GBIF.org on 2022-09-04..
33. Brotto M (2024). MBM herbarium - Museu Botânico Municipal \ Curitiba - Herbário Virtual REFLORA. Version 1.282. Instituto de Pesquisas Jardim Botanico do Rio de Janeiro. Occurrence dataset https://doi.org/10.15468/v52pmc accessed via GBIF.org on 2022-09-04.
34. Bytebier D B, Ranwashe F (2016). NU: KwaZulu-Natal Invasive Alien Species (1821-2011). South African National Biodiversity Institute. Occurrence dataset https://doi.org/10.15468/81w5bx accessed via GBIF.org on 2022-09-04.
35. Chukwuma E, Agbo-Adediran A, Chukwuma D, Ugbogu O (2022). Sampling Event Dataset from Oba Hills, Osun State. Version 1.5. Forestry Research Institute of Nigeria. Sampling event dataset https://doi.org/10.15468/zkkrum accessed via GBIF.org on 2022-09-04.
36. Citizen Science Observation Dataset B, Tiago P (2020). Biodiversity4all Research-Grade Observations. BioDiversity4All. Occurrence dataset https://doi.org/10.15468/njmmp7 accessed via GBIF.org on 2022-09-04.
37. Conselleria de Medio Ambiente, Agua, Infraestructuras y Territorio. Generalitat Valenciana (2022). Banco de Datos de la Biodiversidad de la Comunitat Valenciana. Biodiversity data bank of Generalitat Valenciana. Occurrence dataset https://doi.org/10.15468/b4yqdy accessed via GBIF.org on 2022-09-04.
38. Conservation International Rapid Assessment Program (RAP) Biodiversity Survey Database Occurrence dataset https://doiorg/1015468/tsrjm0 accessed via GBIForg on 2022-09-04.
39. Conservation International. Rapid Assessment Program (RAP) Biodiversity Survey Database. Occurrence dataset https://doi.org/10.15468/tsrjm0 accessed via GBIF.org on 2022-09-04.
40. Costa J C, Capelo J, Jardim R, Sequeira M, Espírito-Santo D, Lousã M, Fontinha S, Aguiar C, Rivas-Martínez S, Tomás D, Coelho P (2021) Catalogue of the plant communities of Madeira and Porto Santo Version 12 Instituto Superior de Agronomia / Universidade de Lisboa Sampling event dataset https://doiorg/1015468/4pey4p accessed via GBIForg on 2022-09-04. Accessed from R via rgbif (https://githubcom/ropensci/rgbif) on 2022-09-04.
41. Crespo M B (2018). Herbario ABH (Universidad de Alicante). Depto. de Ciencias Ambientales y Recursos Naturales, Universidad de Alicante. Occurrence dataset https://doi.org/10.15468/hs9hmr accessed via GBIF.org on 2022-09-04.
42. Crop Wild Relatives Occurrence data consortia (2018) A global database for the distributions of crop wild relatives Version 112 Centro Internacional de Agricultura Tropical - CIAT Occurrence dataset https://doiorg/1015468/jyrthk accessed via GBIForg on 2022-09-04.
43. CSIC-Real Jardín Botánico, Castilla F (2022). CSIC-Real Jardín Botánico-Colección de Plantas Vasculares (MA). Real Jardín Botánico (CSIC). Occurrence dataset https://doi.org/10.15468/mug7kr accessed via GBIF.org on 2022-09-04.
44. de Carvalho e Vasconcelos M T, Santos F, Afonso B (2021). Floristic Study in Orchards - Mafra and Torres Vedras, Portugal. Instituto Superior de Agronomia / Universidade de Lisboa. Sampling event dataset https://doi.org/10.15468/6auk2b accessed via GBIF.org on 2022-09-04.
45. De la Rosa L (2022). INIA-CRF (CSIC) Plant genetic resources for agriculture and food base- collection. Version 1.3. INIA. Centro Nacional de Recursos Fitogenéticos (CSIC). Occurrence dataset https://doi.org/10.15470/lpai7b accessed via GBIF.org on 2022-09-04.
46. Denis Burgos A (2022) Herbario del Jardín Botánico-Histórico La Concepción: HBC Version 16 Concepcion Historical-Botanical Gardens Occurrence dataset https://doiorg/1015468/vfvnmu accessed via GBIForg on 2022-09-04.
47. Dipartimento di Biologia, Università di Pisa (2020). Wikiplantbase #Italia. Occurrence dataset https://doi.org/10.15468/cfhmbw accessed via GBIF.org on 2022-09-04.
48. DOSSA GBO C F, HOUNDONOUGBO A, LUKULA L F (2020). Recensement des espèces en vue de l'élaboration du répertoire des forêts sacrées, forêts galeries, savanes et autres formation floristique du Bénin. ONG Rêve développement. Occurrence dataset https://doi.org/10.15468/hcix0b accessed via GBIF.org on 2022-09-04.
49. Essou F (2020). Diversity and distribution of multipurpose species in Benin. Laboratoire d'Ecologie Appliquée/Université d’Abomey-Calavi (LEA/UAC). Occurrence dataset https://doi.org/10.15468/i7nkmo accessed via GBIF.org on 2022-09-04..
50. Etchike D (2016). Herbier Ecole de Faune. Version 2.1. Ecole de Faune de Garoua. Occurrence dataset https://doi.org/10.15468/jbqjzg accessed via GBIF.org on 2022-09-04..
51. European Bioinformatics Institute (EMBL-EBI), GBIF Helpdesk (2022) INSDC Sequences Version 165 European Nucleotide Archive (EMBL-EBI) Occurrence dataset https://doiorg/1015468/sbmztx accessed via GBIForg on 2022-09-04.
52. European Bioinformatics Institute (EMBL-EBI), GBIF Helpdesk (2022). INSDC Sequences. Version 1.65. European Nucleotide Archive (EMBL-EBI). Occurrence dataset https://doi.org/10.15468/sbmztx accessed via GBIF.org on 2022-09-04.
53. FAVI F, Gbetoho J (2018). Diversité et distribution des espèces de plantes menacées au Bénin. Version 1.1. Faculté des Sciences et Techniques (FAST). Occurrence dataset https://doi.org/10.15468/mpx4qk accessed via GBIF.org on 2022-09-04.
54. Figueira R (2017) IICT Herbário LISC Version 42 Instituto de Investigação Científica Tropical Occurrence dataset https://doiorg/1015468/iinlqm accessed via GBIForg on 2022-09-04.
55. FINGU MABOLA J C, Theeten F (2015) Université Pédagogique Nationale de Kinshasa (UPN) - Plantes fourragères de la République démocratique du Congo - Projet CABIN Université Pédagogique Nationale de Kinshasa Occurrence dataset https://doiorg/1015468/xv4ufh accessed via GBIForg on 2022-09-04. International (CC BY-NC 40)
56. Freeman B (2018). Birds and plants of Firestone Harbel Plantation. Version 1.1. University of Liberia. Occurrence dataset https://doi.org/10.15468/z1qxgu accessed via GBIF.org on 2022-09-04.
57. Fuster Bejarano F, Rita Larrucea J (2022). Herbario de la Universitat de les Illes Balears. Version 1.3. Universitat de les Illes Balears. Occurrence dataset https://doi.org/10.15470/qkd9dg accessed via GBIF.org on 2022-09-04.
58. Fuster Bejarano F, Rita Larrucea J (2022). Herbario de la Universitat de les Illes Balears. Version 1.3. Universitat de les Illes Balears. Occurrence dataset https://doi.org/10.15470/qkd9dg accessed via GBIF.org on 2022-09-04..
59. GAGNIBO C N, Gbètoho A J (2016). Census of plant species in Monts-Kouffè regions, center Benin. Version 1.1. GBIF Benin. Occurrence dataset https://doi.org/10.15468/9tfduu accessed via GBIF.org on 2022-09-04.
60. Gaisberger H, Endresen D (2019). Bioversity Collecting Mission Database. Version 1.10. Bioversity International. Occurrence dataset https://doi.org/10.15468/ulk1iz accessed via GBIF.org on 2022-09-04.
61. Gangnibo C N, Gbètoho A J (2016). Census of species recorded during phytosociological surveys in Benin. Version 1.1. GBIF Benin. Occurrence dataset https://doi.org/10.15468/6habyb accessed via GBIF.org on 2022-09-04.
62. García Sánchez J (2022) MGC Herbarium of University of Malaga (Spain): MGC-Cormof dataset University of Malaga Occurrence dataset https://doiorg/1015468/2gfyxk accessed via GBIForg on 2022-09-04.
63. GBAGUIDI R, KAKPO S B, KOURA K, GANGLO J C (2019) Inventaire des espèces végétales dans le cadre de plusieurs études menées au Sud-Bénin Version 11 Centre d'Etudes de Recherches et de Formation Forestières (CERF) Occurrence dataset https://doiorg/1015468/useh32 accessed via GBIForg on 2022-09-04. Accessed from R via rgbif (https://githubcom/ropensci/rgbif) on 2022-09-04.
64. GBETOHO J (2018). Distribution de quelques espèces végétales du Bénin. Version 1.2. Laboratory of Forest Sciences (University of Abomey-Calavi). Occurrence dataset https://doi.org/10.15468/5j9fd4 accessed via GBIF.org on 2022-09-04.
65. Gil Vives L, Cardona Ametller C V, Ribas-Serra A (2022). Flora vascular de Mallorca, Illes Balears. Version 1.4. Universitat de les Illes Balears. Occurrence dataset https://doi.org/10.15470/bftzuw accessed via GBIF.org on 2022-09-04.
66. Gimenez Luque E (2022). Herbario de la Universidad de Almería. Dept. of Vegetal Biology and Ecology, Faculty of Experimental Science, University of Almeria. Occurrence dataset https://doi.org/10.15468/d5is9n accessed via GBIF.org on 2022-09-04.
67. Gimenez Luque E (2022). Herbario de la Universidad de Almería. Dept. of Vegetal Biology and Ecology, Faculty of Experimental Science, University of Almeria. Occurrence dataset https://doi.org/10.15468/d5is9n accessed via GBIF.org on 2022-09-04.
68. GOULI G Z R, Pagny P J, ASSEH E E, AKAFFOU S V E, TIEBRE M, N'GORAN K S B (2022). Données d'occurrence de quelques espèces végétales de conservation de Côte d'Ivoire. Version 1.3. Centre National de Floristique. Occurrence dataset https://doi.org/10.15468/beb6ek accessed via GBIF.org on 2022-09-04.
69. Houndonougbo A, WIDEDJI J B, KAKPO S B, KOURA K, GANGLO J C (2019) Inventory of plant species through Benin (West Africa) ONG Rêve développement Occurrence dataset https://doiorg/1015468/kiwmh2 accessed via GBIForg on 2022-09-04.
70. HOUNSA M, Gbetoho A (2019). Biodiversité végétale de quelques écosystèmes au Bénin. Laboratory of Forest Sciences (University of Abomey-Calavi). Occurrence dataset https://doi.org/10.15468/a1cdnr accessed via GBIF.org on 2022-09-04.
71. Hsieh C, Lai K (2020). The digitization of plant specimens of NTU. Version 5.2. TELDAP. Occurrence dataset https://doi.org/10.15468/sdyzw5 accessed via GBIF.org on 2022-09-04.
72. iNaturalist contributors, iNaturalist (2022) iNaturalist Research-grade Observations iNaturalistorg Occurrence dataset https://doiorg/1015468/ab3s5x accessed via GBIForg on 2022-09-04.
73. Inventaire National du Patrimoine Naturel (2021) CREATION D’UN INTERNAT ET DES OPERATIONS DIVERSIFIEES AU LYCEE AGRICOLE DE COCONI - Inventaires de la Flore UMS PatriNat (OFB-CNRS-MNHN), Paris Occurrence dataset https://doiorg/1015468/m7aege accessed via GBIForg on 2022-09-04.
74. Jang’andu M, David B, Sigani B, Uisso A, Njovangwa G, Massawe J, Nkya S, Kajembe J (2022). Diversity and composition of preserved angiosperm specimens at Tanzania Forestry Research Institute (TAFORI) Herbarium. Version 1.14. TanBIF. Occurrence dataset https://doi.org/10.15468/sktqsa accessed via GBIF.org on 2022-09-04.
75. Jimu L, Mapaura A, Mureva A, Mujuru L, Muvengwi J, Nyakudya I, Mapaura A (2019) Occurrence data set of Non-Timber Forest Products species for medicinal purposes in Zimbabwe’s biodiversity hotspots Version 12 Bindura University Of Science Education Occurrence dataset https://doiorg/1015468/m2s30i accessed via GBIForg on 2022-09-04.
76. KAKPO S, KOURA K, GANGLO J C (2019) Census of plant species of Benin (West Africa): Database4 SOS Biodiversity Occurrence dataset https://doiorg/1015468/3zjlhv accessed via GBIForg on 2022-09-04.
77. Khaki Mponya N, Maxted N, Magombo Z L K (2022). Diversity and distribution of sorghum wild species in Malawi. Version 1.2. Malawi Plant Genetic Resources Centre (MPGRC). Occurrence dataset https://doi.org/10.15468/pswjzk accessed via GBIF.org on 2022-09-04.
78. Kingbo A, GANGLO J (2017) Census of the species of Benin Data mobilized in the framework of JRS Benin project and funded by JRS Biodiversity Foundation Version 15 Laboratory of Forest Sciences (University of Abomey-Calavi) Occurrence dataset https://doiorg/1015468/uhrfuc accessed via GBIForg on 2022-09-04.
79. KINGBO A, KIKI P P B (2016). Census of the flora of Benin. Version 1.2. GBIF Benin. Occurrence dataset https://doi.org/10.15468/jgauer
80. Kingbo A, Kiki P, Ganglo J (2019). Census of medicinal plants of Benin. Version 1.7. GBIF Benin. Occurrence dataset https://doi.org/10.15468/p4cwmb accessed via GBIF.org on 2022-09-04.
81. KOURA K (2017). Agroforestry species in Parkia biglobosa's parklands in Northern Benin. Laboratory of Forest Sciences (University of Abomey-Calavi). Occurrence dataset https://doi.org/10.15468/iokuu0 accessed via GBIF.org on 2022-09-04.
82. Kukk T. Estonian University of Life Sciences Institute of Agricultural and Environmental Sciences Vascular Plant Herbarium. Estonian University of Life Sciences. Occurrence dataset https://doi.org/10.15468/m3x9uu accessed via GBIF.org on 2022-09-04.
83. Laghetti G, Scarascia M, Cataldo P, Stimolo L, De Paola D, Direnzo P, Rapanà N, Vendramin G G, Urbano M, Bucci G, Ianigro M (2022). Mediterranean Germplams Genebank - Miscellaneous species (ITA436-MGG-Miscellaneous). Consiglio Nazionale delle Ricerche, Istituto di Bioscienze e BioRisorse (CNR-IBBR). Occurrence dataset https://doi.org/10.15468/rpej8x accessed via GBIF.org on 2022-09-04.
84. López Martínez M, Martín-Consuegra E (2021). Jardín Botánico de Córdoba: Herbarium COA. Botanical Garden of Córdoba. Occurrence dataset https://doi.org/10.15468/vjuvw1 accessed via GBIF.org on 2022-09-04.
85. Madrigal O (2012) Wilson Botanical Garden - Las Cruces Biological Station Organization for Tropical Studies Occurrence dataset https://doiorg/1015468/xxrs0v accessed via GBIForg on 2022-09-04.
86. Madrigal O (2012). Wilson Botanical Garden - Las Cruces Biological Station. Organization for Tropical Studies. Occurrence dataset https://doi.org/10.15468/xxrs0v accessed via GBIF.org on 2022-09-04.
87. Magassouba S (2019). Herbier National de Guinée (HNG). Version 1.2. Herbier National de Guinée (HNG). Occurrence dataset https://doi.org/10.15468/vnatbk accessed via GBIF.org on 2022-09-04.
88. Magassouba S, Sow B, Guilavogui P, Condé N, Doré T S (2017). La liste Rouge des plantes menacées de la Guinée (BID-AF2015-0042-NAC). Herbier National de Guinée (HNG). Occurrence dataset https://doi.org/10.15468/rqn53h accessed via GBIF.org on 2022-09-04.
89. Masinde S, Kamau P, Odhiambo K (2021). Plant species occurrence in the upper to mid Tana River Basin based on specimen records at the East African Herbarium (EA), Nairobi, Kenya. Version 1.2. National Museums of Kenya. Occurrence dataset https://doi.org/10.15468/5g8b2c accessed via GBIF.org on 2022-09-04.
90. Masinde S, Matheka K, Atieno D, Odhiambo K, Nyange M, Kamau P (2019). Plant species occurrences recorded from selected sites in the upper Tana River Basin, Kenya. National Museums of Kenya. Occurrence dataset https://doi.org/10.15468/a5iokr accessed via GBIF.org on 2022-09-04.
91. Medina Domingo L, Aedo Pérez C (2021). CSIC-Real Jardín Botánico-Anthos. Sistema de Información de las Plantas de España. Version 1.19. Real Jardín Botánico (CSIC). Occurrence dataset https://doi.org/10.15468/4wnutv accessed via GBIF.org on 2022-09-04.
92. Meerts P, Heughebaert A (2019). Herbarium of the Université Libre de Bruxelles. Université Libre de Bruxelles (ULB). Occurrence dataset https://doi.org/10.15468/yn20mq accessed via GBIF.org on 2022-09-04.
93. Meise Botanic Garden (2022) Meise Botanic Garden Herbarium (BR) Version 129 Meise Botanic Garden Occurrence dataset https://doiorg/1015468/wrthhx accessed via GBIForg on 2022-09-04.
94. Menting F (2022). Centre for Genetic Resources, the Netherlands, PGR passport data. Centre for Genetic Resources, The Netherlands. Occurrence dataset https://doi.org/10.15468/mugslo accessed on GBIF.org on 2022-09-04..
95. MGnify (2018). Effect of genetics and environment on gut bacteria in Anopheles and Aedes mosquitoes. Sampling event dataset https://doi.org/10.15468/vktdke accessed via GBIF.org on 2022-09-04.
96. MGnify (2019). Nutrient addition mesocosm experiment Targeted Locus (Loci). Sampling event dataset https://doi.org/10.15468/xjefiq accessed via GBIF.org on 2022-09-04..
97. MGnify (2022). Amplicon sequencing of Tara Oceans DNA samples corresponding to size fractions for protists. Sampling event dataset https://doi.org/10.15468/2hv1be accessed via GBIF.org on 2022-09-04.
98. Misa Aboagye L, Osei-Kofi P S (2022). Wild Crop Relative germplasm collections at Bunso in the Eastern Region of Ghana. Version 1.3. Conservation Alliance International. Occurrence dataset https://doi.org/10.15468/zt6gk4 accessed via GBIF.org on 2022-09-04.
99. Mohamed Vall A, Heughebaert A (2014). Herbier National de Mauritanie (HNM). Ecole Normale Supérieure de Nouakchott. Occurrence dataset https://doi.org/10.15468/mz2hp2 accessed via GBIF.org on 2022-09-04.
100. Moracho E, Calvo G, Gómez J M, Homet P, Rodríguez-Sánchez F, Villalva P, Jordano P (2022). A literature-based inventory of ecological interactions in Doñana National Park. Estación Biológica de Doñana (CSIC). Sampling event dataset https://doi.org/10.15470/jlhz16 accessed via GBIF.org on 2022-09-04..
101. Moussa D, Doumbouya S (2018) Inventaire des espèces végétales productives dans les Systèmes Agro-forestiers aux alentours de la Forêt classée de Ziama en Guinée Forestière (BID-AF2015-0066-REG) Version 13 Institut de Recherche Agronomique de Guinée (IRAG) Occurrence dataset https://doiorg/1015468/c4tz9x accessed via GBIForg on 2022-09-04.
102. Museo Nacional de Costa Rica. herbario. Occurrence dataset https://doi.org/10.15468/yhvbj8 accessed via GBIF.org on 2022-09-04.
103. National Museum of Natural History, Luxembourg (2022). Biodiversity weekend observations, Recorder-Lux database. Version 1.6. Occurrence dataset https://doi.org/10.15468/grbhmn accessed via GBIF.org on 2022-09-04.
104. National Museum of Natural History, Luxembourg (2022). Collections and observation data National Museum of Natural History Luxembourg. Occurrence dataset https://doi.org/10.15468/s2iu7d accessed via GBIF.org on 2022-09-04.
105. Natural History Museum (2022) Natural History Museum (London) Collection Specimens Occurrence dataset https://doiorg/105519/qdjebc7ijb accessed via GBIForg on 2022-09-04.
106. Natural History Museum (2022). Natural History Museum (London) Collection Specimens. Occurrence dataset https://doi.org/10.5519/qd.jebc7ijb accessed via GBIF.org on 2022-09-04.
107. N'GORAN B K S, YAO K, BARIMA S S Y, KOUASSI F A, TRA BI F B, BAKAYOKO A, AKE-ASSI A E, TIEBRE M S, OUATTARA D, N'Goran B (2020) Les spermaphytes de l'Herbier du Centre National de Floristique de Côte d'Ivoire: quatrième partie Version 12 Centre National de Floristique Occurrence dataset https://doiorg/1015468/ekrbpi accessed via GBIForg on 2022-09-04.
108. Nkemehule F, Onuminya T, Ogundipe O (2022). Herbarium specimens held at the College of Medicine, University of Lagos, Lagos, Nigeria. Version 1.3. University of Lagos. Occurrence dataset https://doi.org/10.15468/5ngcgq accessed via GBIF.org on 2022-09-04.
109. Nnabude P, Egboka T, Ugbogu O, Osiyemi O (2019). Plants Of Nnamdi Azikiwe University Herbarium (NAUH) Awka. Version 1.5. Forestry Research Institute of Nigeria. Occurrence dataset https://doi.org/10.15468/qqzpfc accessed via GBIF.org on 2022-09-04.
110. Observation.org (2022). Observation.org, Nature data from around the World. Occurrence dataset https://doi.org/10.15468/5nilie accessed via GBIF.org on 2022-09-04..
111. Odorico D (2021). Colecção Botânica do Instituto de Investigação Agrária de Moçambique. Version 1.13. Herbarium LMA: Agricultural Research Institute of Mozambique. Occurrence dataset https://doi.org/10.15468/hsez7x accessed via GBIF.org on 2022-09-04.
112. Oliveira R C D, Cemin D (2022). UB herbarium - Universidade de Brasília - Herbário Virtual REFLORA. Version 1.206. Instituto de Pesquisas Jardim Botanico do Rio de Janeiro. Occurrence dataset https://doi.org/10.15468/x6pmvz accessed via GBIF.org on 2022-09-04.
113. Onuminya T, Ogundipe O (2022): Flora of South-Southern Nigeria Coastal Environments. v1.2. University of Lagos. Dataset/Occurrence. https://ipt-nigeria.gbif.fr/resource?r=ssflora&v=1.2 https://doi.org/10.15468/wxak7w accessed via GBIF.org on 2022-09-04.
114. Onuminya T, Ogundipe O, Igbari A (2022). A record of Plants used in Treating Dental Diseases in Lagos Nigeria. Version 1.1. University of Lagos. Occurrence dataset https://doi.org/10.15468/bygfuz accessed via GBIF.org on 2022-09-04.
115. Onuminya T, Ogundipe O, Igbari A, Kemabonta K (2022). Higher Plant Collections of the Lagos University Herbarium (LUH): Families A - Z. Version 1.5. University of Lagos. Occurrence dataset https://doi.org/10.15468/ty22qw accessed via GBIF.org on 2022-09-04.
116. Osei-Kofi P S, Misa Aboagye L, Mbawine J S (2018). Root and Tuber germplasm collections at Bunso in the Eastern Region of Ghana. A Rocha Ghana. Occurrence dataset https://doi.org/10.15468/r69kmj accessed via GBIF.org on 2022-09-04.
117. OYEDOKOU E A E N, HOUNSA M A F (2020). Plant spicies from Mono Delta transboundary biosphere reserve. Faculté des Sciences et Techniques (FAST). Occurrence dataset https://doi.org/10.15468/dj9mv4 accessed via GBIF.org on 2022-09-04.
118. Paul Smart O (2021). Foreign plant germplasm collections in Ghana. Version 1.3. Council for Scientific and Industrial Research-Plant Genetic Resources Research Institute (CSIR-PGRRI),Ghana. Occurrence dataset https://doi.org/10.15468/1pjfla accessed via GBIF.org on 2022-09-04.
119. Pescott O L, Peyton J M, Mountford J O, Pescott O (2020). UKSBA Cyprus Plant Records 2018. Version 1.2. Biological Records Centre. Occurrence dataset https://doi.org/10.15468/xp6bam accessed via GBIF.org on 2022-09-04..
120. Pescott O, Peyton J, Mountford J O (2020). UKSBA Cyprus - Habitat Samples, 2019. Version 1.1. Biological Records Centre. Sampling event dataset https://doi.org/10.15468/c8p4qe accessed via GBIF.org on 2022-09-04.. Accessed from R via rgbif
121. Pirani J R, Jono V, Lohmann L G, Figueiredo R R D (2024). SPF herbarium - Universidade de são Paulo - Herbário Virtual REFLORA. Version 1.269. Instituto de Pesquisas Jardim Botanico do Rio de Janeiro. Occurrence dataset https://doi.org/10.15468/nt6dng accessed via GBIF.org on 2022-09-04.
122. Rabarivola M L, Razafiniary V, Razanajatovo H, Rapanarivo S H, Ralimanana H, Vorontsova M (2019). All herbarium specimens of grasses held at TAN herbarium. Version 1.8. Kew Madagascar Conservation Centre - Royal Botanic Gardens Kew. Occurrence dataset https://doi.org/10.15468/v6mfx2 accessed via GBIF.org on 2022-09-04.
123. Rabarivola M L, Razanajatovo H, Razafiniary V, Ralimanana H, Vorontsova M (2019). Research database of Madagascar grasses compiled by Maria Vorontsova. Version 1.3. Kew Madagascar Conservation Centre - Royal Botanic Gardens Kew. Occurrence dataset https://doi.org/10.15468/umduhk accessed via GBIF.org on 2022-09-04.
124. RADJI R P, AKPENE K (2018). Liste des récoltes par Herbier de dépôt TOGO. Version 1.3. Université de Lomé. Occurrence dataset https://doi.org/10.15468/5wurxy accessed via GBIF.org on 2022-09-04.
125. Radji R P, AKPENE K (2018). Togo National Herbarium Database. Version 3.8. Université de Lomé. Occurrence dataset https://doi.org/10.15468/h3mptq accessed via GBIF.org on 2022-09-04.
126. Radji R P, Magassouba S, Engone Obiang N L, Betti J L, Koffi K J, Yedomonhan H (2022) Collections des Herbiers d’Afrique de l'Ouest et d'Afrique Centrale Version 13 Université de Lomé Occurrence dataset https://doiorg/1015468/tn8tu9 accessed via GBIForg on 2022-09-04.
127. RAKOTONDRAJAONA R, RAKOTOARISOA M, Ranaivo J, RAKOTONANDRASANA S, RANDRIAMBANONA H (2018). Database on reference specimens for medicinal plants of the Euphorbiaceae family, conserved at the CNARP herbarium. Version 1.3. Madagascar Biodiversity Information Facility (MadBIF). Occurrence dataset https://doi.org/10.15468/eabysc accessed via GBIF.org on 2022-09-04.
128. Ranatunga D, Hoare B (2022). Auckland Museum Botany Collection. Version 1.94. Auckland War Memorial Museum. Occurrence dataset https://doi.org/10.15468/mnjkvv accessed via GBIF.org on 2022-09-04.
129. Ranwashe F (2022) Botanical Database of Southern Africa (BODATSA): Botanical Collections Version 121 South African National Biodiversity Institute Occurrence dataset https://doiorg/1015468/2aki0q accessed via GBIForg on 2022-09-04.
130. Ranwashe F (2022). Botanical Database of Southern Africa (BODATSA): Botanical Collections. Version 1.21. South African National Biodiversity Institute. Occurrence dataset https://doi.org/10.15468/2aki0q accessed via GBIF.org on 2022-09-04.
131. Riera Vicent J (2022). Colección de plantas vasculares del herbario de la Universitat de València (VAL). Botanical Garden, University of Valencia. Occurrence dataset https://doi.org/10.15468/xmki52 accessed via GBIF.org on 2022-09-04.
132. ROBERT S, LEPAREUR F, Inventaire National du Patrimoine Naturel (2022) Données d'occurrences Espèces issues de l'inventaire des ZNIEFF Version 17 UMS PatriNat (OFB-CNRS-MNHN), Paris Occurrence dataset https://doiorg/1015468/ikshke accessed via GBIForg on 2022-09-04. Accessed from R via rgbif (https://githubcom/ropensci/rgbif) on 2022-09-04.
133. Rodríguez Riaño T (2022). Herbarium of Vascular Plants Collection of the University of Extremadura (Spain). Dep. of Plant Biology, Ecology and Earth Sciences, Botany Area, Univ. Extremadura. Occurrence dataset https://doi.org/10.15468/l8vpay accessed via GBIF.org on 2022-09-04.
134. Rodríguez Riaño T (2022). Herbarium of Vascular Plants Collection of the University of Extremadura (Spain). Dep. of Plant Biology, Ecology and Earth Sciences, Botany Area, Univ. Extremadura. Occurrence dataset https://doi.org/10.15468/l8vpay accessed via GBIF.org on 2022-09-04.
135. Romero-Molina J M, Benítez-Cruz G, Jiménez-Olivencia Y, González-Tejero García M R, Molero-Mesa J, Ibáñez-Jiménez Á J, Porcel-Rodríguez L (2022). Colección de semillas de variedades locales de la Alpujarra granadina. Version 1.1. Sierra Nevada Global Change Observatory. Andalusian Environmental Center, University of Granada, Regional Government of Andalusia. Occurrence dataset https://doi.org/10.15470/zsrwz2 accessed via GBIF.org on 2022-09-04.
136. Royal Botanic Gardens, Kew (2021). Royal Botanic Gardens, Kew - Herbarium Specimens. Occurrence dataset https://doi.org/10.15468/ly60bx accessed via GBIF.org on 2022-09-04.
137. Sales F, Santos J (2024). Herbarium of University of Coimbra (COI). Version 1.70. Herbarium of University of Coimbra (COI). Occurrence dataset https://doi.org/10.15468/7x9xtx accessed via GBIF.org on 2022-09-04.
138. Sánchez Gómez P (2020). Herbario de Universidad de Murcia: MUB. Department of Plant Biology. Faculty of Biological Sciences. Univ. Murcia. Occurrence dataset https://doi.org/10.15468/qnv1aq accessed via GBIF.org on 2022-09-04.
139. Sawe T, Kaniki A, Gideon H, Kissima A, Munishi P, Mbwambo J, Jang’andu M, David B, Sigani B, Uisso A, Njovangwa G, Massawe J, Nkya S, Kajembe J (2022). Diversity and composition of preserved angiosperm specimens at Tanzania Forestry Research Institute (TAFORI) Herbarium. Version 1.14. TanBIF. Occurrence dataset https://doi.org/10.15468/sktqsa accessed via GBIF.org on 2022-09-04.
140. Scarcelli N (2021). Occurrence of wild and cultivated yams observed in West Africa and Madagascar from 1996 to 2015. Version 1.1. IRD - Institute of Research for Development. Occurrence dataset https://doi.org/10.15468/4ma2j7 accessed via GBIF.org on 2022-09-04.
141. Senckenberg (2020) African Plants - a photo guide Occurrence dataset https://doiorg/1015468/r9azth accessed via GBIForg on 2022-09-04.
142. Seychelles Key Biodiversity Areas National Coordination Group, Senterre B (2022). Checklist and distribution of the species of Seychelles for conservationists. Version 1.9. Seychelles National Herbarium. Checklist dataset https://doi.org/10.15468/rezu4h accessed via GBIF.org on 2022-09-04.
143. Sharaibi O, Oluwa O, Omolokun K, Adebayo A, Mamudu M, Onuminya T (2022) Plant Collections in the Lagos State University Herbarium, Ojo, Lagos, Nigeria Lagos State University Occurrence dataset https://doiorg/1015468/b67erz accessed via GBIForg on 2022-09-04.
144. Sidibe S, Akbaraly M (2016) Phanérogames recensés aux Monts Nimba Version 31 Centre d'Observation de Surveillance et d'Information Environnementales (COSIE) Occurrence dataset https://doiorg/1015468/nu7rmc accessed via GBIForg on 2022-09-04.
145. Soudzilovskaia N A, Vaessen S, Barcelo M, He J, Rahimlou S, Abarenkov K, Brundrett M C, Gomes S, Merckx V, Martinez-Suz L, Tedersoo L. Taxon occurrence data for the FungalRoot database. PlutoF. Occurrence dataset https://doi.org/10.15468/a7ujmj accessed via GBIF.org on 2022-09-04.
146. Souza M (2024). HUEM herbarium - Universidade Estadual de Maringá - Herbário Virtual REFLORA. Version 1.129. Instituto de Pesquisas Jardim Botanico do Rio de Janeiro. Occurrence dataset https://doi.org/10.15468/r35dtr accessed via GBIF.org on 2022-09-04.
147. Staatliche Naturwissenschaftliche Sammlungen Bayerns. Fungus Collections at Staatliches Museum für Naturkunde Karlsruhe (Herbarium KR). Occurrence dataset https://doi.org/10.15468/0bhhip accessed via GBIF.org on 2022-09-04.
148. Taugourdeau S, Daget P (2019). FLOTROP, a massive contribution to plant diversity data for open ecosystems in Tropical Africa. Version 1.5. CIRAD SELMET. Occurrence dataset https://doi.org/10.15468/oxunf1 accessed via GBIF.org on 2022-09-04.
149. Teisher J, Stimmel H (2024) Tropicos MO Specimen Data Missouri Botanical Garden Occurrence dataset https://doiorg/1015468/hja69f accessed via GBIForg on 2022-09-04.
150. Tela Botanica Carnet en Ligne Occurrence dataset https://doiorg/1015468/rydcn2 accessed via GBIForg on 2022-09-04.
151. The International Barcode of Life Consortium (2024) International Barcode of Life project (iBOL) Occurrence dataset https://doiorg/1015468/inygc6 accessed via GBIForg on 2022-09-04.
152. TOGNI Y (2017). Data collected in the framework of the accomplishment of research activities. Laboratory of Forest Sciences (University of Abomey-Calavi). Occurrence dataset https://doi.org/10.15468/blra4p accessed via GBIF.org on 2022-09-04.
153. TOHINNOU HOUEZE C V G (2020). Floristic diversity of the protected areas of Mont Kouffé and Wari Maro in the north of Benin. Laboratoire d'Ecologie Appliquée/Université d’Abomey-Calavi (LEA/UAC). Sampling event dataset https://doi.org/10.15468/qy58kd accessed via GBIF.org on 2022-09-04.
154. Tushabe D H (2021) Data from EIAs and other studies coordinated by the national forestry authority of Uganda Version 14 National Biodiversity Data Bank Occurrence dataset https://doiorg/1015468/ku21hg accessed via GBIForg on 2022-09-04.
155. Ugbogu O, Osiyemi O, Adeniji K, Okanlawo T, Soyewo T, Oyebola T, Chukwuma E, Aina S, Odewo A (2019). Grasses of the Forest Herbarium Ibadan (FHI) Nigeria. Version 1.8. Forestry Research Institute of Nigeria. Occurrence dataset https://doi.org/10.15468/rhbyxz accessed via GBIF.org on 2022-09-04.
156. UMR AMAP. WIKTROP - Weed Identification and Knowledge in the Tropical and Mediterranean areas. Occurrence dataset https://doi.org/10.15468/dvc7wm accessed via GBIF.org on 2022-09-04.
157. University of Calabar: Biodiversity of Onne Local Government Area, Rivers State, Nigeria https://doi.org/10.15468/yyapgv accessed viaGBIF.org on 2022-09-04.
158. University of California Riverside (2024). UCR - University of California, Riverside Herbarium - Vascular Plants. Occurrence dataset https://doi.org/10.15468/ai1kou accessed via GBIF.org on 2022-09-04.
159. University of Graz, Institute of Plant Sciences. University of Graz, Institute of Plant Sciences - Herbarium GZU. Occurrence dataset https://doi.org/10.15468/axtkuz accessed via GBIF.org on 2022-09-04.
160. University of Oslo (2024). Vascular Plant Herbarium, Oslo (O) UiO. Version 1.2182. Occurrence dataset https://doi.org/10.15468/wtlymk accessed via GBIF.org on 2022-09-04.
161. University of Vienna - Herbarium WU. University of Vienna, Institute for Botany - Herbarium WU. Occurrence dataset https://doi.org/10.15468/tnj8wm accessed via GBIF.org on 2022-09-04.
162. US National Plant Germplasm System. United States National Plant Germplasm System Collection. Occurrence dataset https://doi.org/10.15468/ce7fox accessed via GBIF.org on 2022-09-04.
163. Uva J S, Onofre R, Moreira J, Faias S P, Barreiro S, Santos E, Capelo J, Corte-Real L, Martins J, Ribeiro J R, Cancela J, Rainha M, Amaral N, Santos C, Perpétua J, Pinho J, Araújo J M, Reis L, Canaveira P, Paulino J, Pina A, Binev Y, Coelho P (2021). Forestry Inventory 2015. ICNF - Instituto da Conservação da Natureza e das Florestas. Sampling event dataset https://doi.org/10.15468/33hvm4 accessed via GBIF.org on 2022-09-04..
164. Van der Bank, Ranwashe F (2019) FBIP: Magnoliophyta collection Version 11 South African National Biodiversity Institute Occurrence dataset https://doiorg/1015468/x4hw9t accessed via GBIForg on 2022-09-04.
165. Vanderplank S, Comisión nacional para el conocimiento y uso de la biodiversidad C (2022). Puntos de presencia en Baja California (Botany). Version 1.7. Comisión nacional para el conocimiento y uso de la biodiversidad. Occurrence dataset https://doi.org/10.15468/87mubt accessed via GBIF.org on 2022-09-04.
166. Vázquez Pardo F M, Márquez García F (2021). Herbario HSS Finca La Orden-Valdesequera (CICYTEX). Junta de Extremadura. La Orden Estate, Dep. Forest Production and Grasslands, Agriculture and Environment Office, Regional Government of Extremadura. Occurrence dataset https://doi.org/10.15468/siye1z accessed via GBIF.org on 2022-09-04.
167. Vicens M (2022). Hortus Botanicus Sollerensis Herbarium (FBonafè). Sóller Botanical Garden Foundation. Occurrence dataset https://doi.org/10.15468/ghnxdi accessed via GBIF.org on 2022-09-04.
168. Vizoso Paz M T (2022). Herbario de la Universidad de Granada: Colección GDA-Fanerogamia. Version 1.8. Herbario de la Universidad de Granada. Occurrence dataset https://doi.org/10.15468/pyzji3 accessed via GBIF.org on 2022-09-04.
169. wafongo E (2022). A checklist of plant species observed in some parts of a degraded Thyolo mountain forest Reserve. Version 1.33. National Herbarium & Botanic Gardens of Malawi. Occurrence dataset https://doi.org/10.15468/hnvndq accessed via GBIF.org on 2022-09-04.
170. Xavier Font (2022). Sistema de Información de la vegetación Ibérica y Macaronésica. Version 1.6. Banc de dades de biodiversitat de Catalunya. Occurrence dataset https://doi.org/10.15468/qyzfdt accessed via GBIF.org on 2022-09-04.
171. Yang Z, Xu Z (2019). Some Plant Specimens from KUN, IBSC, NAS Herbarium in China from 1900 to 1950. Chinese Academy of Sciences (CAS). Occurrence dataset https://doi.org/10.15468/irnwew accessed via GBIF.org on 2022-09-04.
172. YAO K, KOUASSI A F, TRA BI F B, BARIMA S Y S, AKPATOU K B, BAKAYOKO A, AKE-ASSI A E, TIEBRE M S, OUATTARA D, N'GORAN B (2020). Les spermaphytes de l'Herbier du Centre National de Floristique de Côte d'Ivoire: troisième partie. Version 1.2. Centre National de Floristique. Occurrence dataset https://doi.org/10.15468/3vxxjd accessed via GBIF.org on 2022-09-04.
173. Yêhouénou Tessi D R (2017). Census of medicinal and agroforestry plants of Benin. Laboratory of Forest Sciences (University of Abomey-Calavi). Occurrence dataset https://doi.org/10.15468/y8jm57 accessed via GBIF.org on 2022-09-04.
174. YEVIDE A I, GANGLO J C (2016). Ecology, structure and dynamics of private plantations of Teak (Tectona grandis Lf) of the Department of the Atlantic (South Benin), 2011. Version 1.3. GBIF Benin. Occurrence dataset https://doi.org/10.15468/fkiroj accessed via GBIF.org on 2022-09-04.
175. Zyrdal M (2022) Botany (UPS) Museum of Evolution, Uppsala University Occurrence dataset https://doiorg/1015468/ufmslw accessed via GBIForg on 2022-09-04.
